# Rh2E2, a novel metabolic suppressor, specifically inhibits energy-based metabolism of tumor cells

**DOI:** 10.18632/oncotarget.6934

**Published:** 2016-01-18

**Authors:** Vincent Kam Wai Wong, Hang Dong, Xu Liang, Li-Ping Bai, Zhi-Hong Jiang, Yue Guo, Ah-Ng Tony Kong, Rui Wang, Richard Kin Ting Kam, Betty Yuen Kwan Law, Wendy Wen Luen Hsiao, Ka Man Chan, Jingrong Wang, Rick Wai Kit Chan, Jianru Guo, Wei Zhang, Feng Gen Yen, Hua Zhou, Elaine Lai Han Leung, Zhiling Yu, Liang Liu

**Affiliations:** ^1^ State Key Laboratory of Quality Research in Chinese Medicine, Macau University of Science and Technology, Taipa, Macau, China; ^2^ Shum Yiu Foon Shum Bik Chuen Memorial Centre for Cancer and Inflammation Research, School of Chinese Medicine, Hong Kong Baptist University, Kowloon Tong, Hong Kong, China; ^3^ Department of Pharmaceutics, Ernest Mario School of Pharmacy, Rutgers, the State University of New Jersey, Piscataway, New Jersey, USA

**Keywords:** Rh2E2, metabolic suppressor, alpha-enolase, energy metabolism, anti-tumor

## Abstract

Energy metabolism in cancer cells is often increased to meet their higher proliferative rate and biosynthesis demands. Suppressing cancer cell metabolism using agents like metformin has become an attractive strategy for treating cancer patients. We showed that a novel ginsenoside derivative, Rh2E2, is as effective as aspirin in preventing the development of AOM/DSS-induced colorectal cancer and suppresses tumor growth and metastasis in a LLC-1 xenograft. A sub-chronic and acute toxicity LD_50_ test of Rh2E2 showed no harmful reactions at the maximum oral dosage of 5000 mg/kg body weight in mice. Proteomic profiling revealed that Rh2E2 specifically inhibited ATP production in cancer cells via down-regulation of metabolic enzymes involving glycolysis, fatty acid β-oxidation and the tricarboxylic acid cycle, leading to specific cytotoxicity and S-phase cell cycle arrest in cancer cells. Those findings suggest that Rh2E2 possesses a novel and safe anti-metabolic agent for cancer patients by specific reduction of energy-based metabolism in cancer cells.

## INTRODUCTION

^18^F-deoxyglucose positron emission tomography (FDG-PET) is often used to visualize the increased glucose uptake in tumors of patients [1]. The proliferating cancer cells exhibit a higher cell metabolism compared to most normal differentiated cells [2]. To adapt to the higher rate of proliferation and division of cancer cells, demand of the additional nutrients and sufficient energy are required [3]. Studies of metabolic alteration and adaptation of cancer cells over the past century [4] revealed that glycolysis and glutaminolysis are the major enhanced metabolic pathways for tumor growth and survival [5]. The inhibition of metabolic status of tumor cells are multi-dimensional, such as reduced enzyme expression (e.g. aldolase A, α-enolase, lactate dehydrogenase and fatty acid synthase) [6, 7], mutation (such as isocitrate dehydrogenase (IDH)1/2) [8, 9], post-translational inactivation of pyruvate dehydrogenase kinase [10], and substitution of a different enzyme isoform (e.g., the pyruvate kinase M2 isoform, PKM2) [11]. Therefore, inhibiting multiple mechanisms to reduce or reverse the abnormal reprogrammed metabolism of cancer cells, particularly reducing energy-based metabolism, is an attractive therapeutic strategy for cancer patients [3].

However, inhibition of glycolysis produced limited effect on tumorigenesis because anti-glycolysis treatment could drive tumor cells to demand more glutamine for ATP production via glutaminolysis [12]. Therefore, a new generation of therapeutics, network-based holistic intervention via suppressing the overall metabolism of cancer cells and blocking fuel uptake and ATP production is desirable [13]. The marketed drug metformin exhibits a reliable anti-tumor effect in both pre-clinical and clinical trials via multi-pathway suppressions on cancer cell metabolism including suppressing ATP production through the inhibition of the mitochondrial respiratory chain (complex I) and fatty acid oxidation [14, 15]. Metformin can shut down the external and internal glucose supply to cancer cells by enhancing insulin-mediated peripheral glucose uptake and inhibiting hepatic gluconeogenesis [15, 16]. However, blockage of gluconeogenesis by metformin may produce potential adverse effects like lactic acidosis [17]. Thus, identifying an ideal energy-based metabolic suppressor that specifically reverses the reprogrammed metabolic status of cancer cells but not the cell metabolism of normal tissues, is in demand.

Ginsenoside Rh2 (Rh2) could reduce cell proliferation and induce cell cycle arrest, cell differentiation and apoptosis in variety types of cancer cells, while 20(*R*)-Rh2 exhibited no cytotoxic effect on cancer cells [18–20]. Here, we identified a new ginsenoside derivative, Rh2E2 modified from 20(*R*)-Rh2, has a potent anti-cancer effect in both *in vitro* and *in vivo* experiments, but with less toxicity on normal cells and animals. Rh2E2 was unraveled to suppress tumor growth via down-regulation of metabolic enzymes for energy production; suppression of oncogenic proteins for cancer cell invasion, metastasis, proliferation and cell cycle progression; and activation of ERK-p53/-Egr1 signaling and inhibition of the Skp2 autoinduction loop for cell cycle arrest. Accordingly, Rh2E2 shows valuable as a therapeutic inhibitor of metabolism for treating cancer patients.

## RESULTS

### Rh2E2 possesses a specific cytotoxic effect on cancer cells

**Figure 1:**
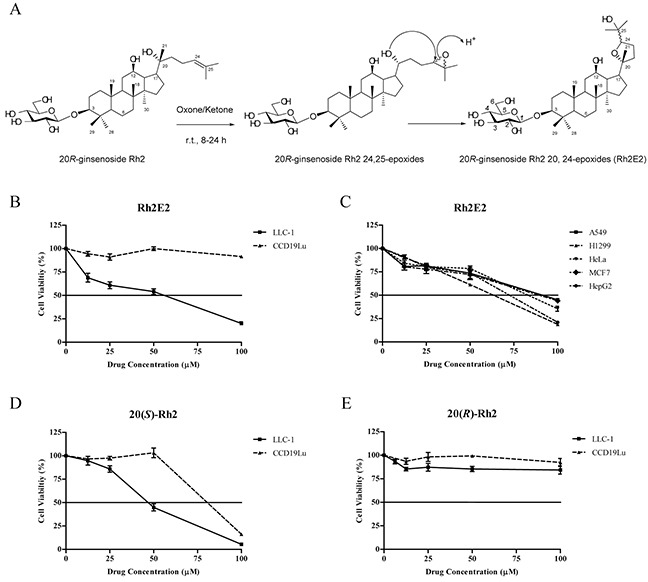
Specific cytotoxicity induced by Rh2E2 in cancer cell cultures **A.** Organic synthesis of Rh2E2. **B.** Cytotoxic effects of Rh2E2 on mouse lung cancer cells (LLC-1) and human normal lung fibroblasts (CCD19Lu). **C.** Cytotoxic effects of Rh2E2 toward a panel of cancer cells from different origins. **D.** Cytotoxic effects of 20(*S*)-Rh2 on LLC-1 and CCD19Lu cells. **E.** Cytotoxic effects of 20(*R*)-Rh2 on LLC-1 and CCD19Lu cells. Cell cytotoxicity was measured by MTT assay after 72 hr incubation. Representative results were shown as Means ± S.E.M. from 3 independent experiments.

The anti-cancer property of Rh2 was only known for 20*S*-ginsenoside Rh2 (20(*S*)-Rh2), but not 20*R*-ginsenoside Rh2 (20(*R*)-Rh2) [18, 19]. However, the poor solubility of 20(*S*)-Rh2 limits its value as a potential anti-tumor agent. We therefore tried to modify its chemical structure of 20(*R*)-Rh2 so as to improve its solubility and enhance its anti-cancer potency. As shown in Figure 1A, the double bond on the side chain of 20(*R*)-Rh2 was oxidized with oxone/NaHCO_3_ in the presence of a ketone catalyst, forming 24, 25-epoxy compounds [21]. The hydroxyl group located at C-20 of these epoxides attacked the electron-demand C-24 to form 20, 24-epoxides in a 1:1 mixture of 24-epimers [22], which was validated by UPLC-MS analysis and purified by ODS column chromatography (90% methanol). The structure of Rh2E2 was characterized by positive high-resolution ESI-MS and its molecular formula is C_36_H_62_O_9_.

As shown in Figure 1B, Rh2E2 exhibited a specific cytotoxic effect on LLC-1 lung cancer cells, with a mean IC_50_ of 56 μM, while it showed no cytotoxic effect on CCD19Lu human normal lung fibroblasts at doses over 100 μM. Also, Rh2E2 demonstrated a dose-dependent cytotoxic effect against other cancer cell lines (Figure 1C). However, 20(*S*)-Rh2, but not 20(*R*)-Rh2 exhibited cytotoxicity both in cancerous and normal cells (Figure 1D & 1E). These results indicate that structural modification of the non-toxic ginsenoside 20(*R*)-Rh2 into the ginsenoside Rh2E2 has induced a change of its cytotoxic properties; i.e., from a non-specific into a specific property in cancer cells.

### Rh2E2 exhibits a chemopreventive effect against AOM/DSS-induced colorectal cancer in mice

**Figure 2:**
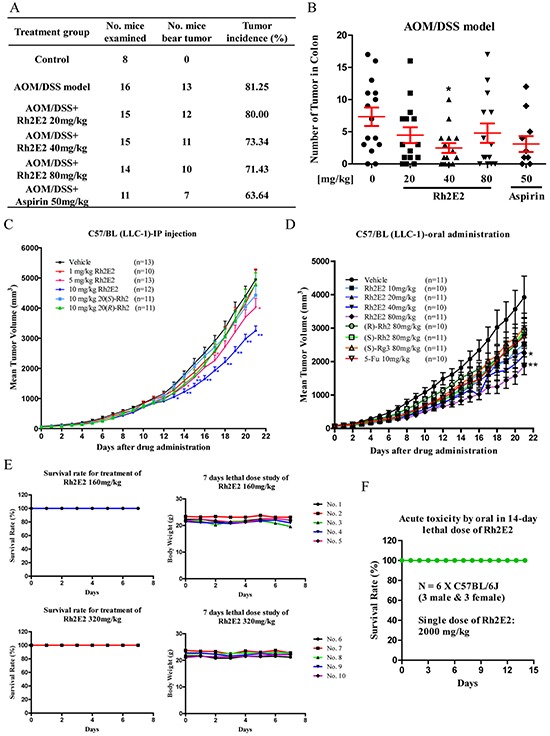
*In vivo* anti-tumor effect of Rh2E2 on AOM/DSS-induced colon carcinogenesis and LLC-1 xenograft mouse model **A.** Effect of Rh2E2 on tumor incidence in AOM/DSS CRC model. Rh2E2 (20, 40 and 80 mg/kg/day) or positive control drug aspirin (50 mg/kg/day) dissolved in PEG400: Ethanol: water = 6:1:3 (v/v/v) was given to the mice by oral gavage administration for 11 weeks. The tumor incidence (%) of each group was calculated from the number of mice with developed colon cancer over the total number of mice examined. **B.** Effect of Rh2E2 on the number of tumors found in mouse colon. At necropsy, the mice colon were dissected longitudinally and the number of tumors were counted together with the volume of tumor in length (mm) × width (mm) × height (mm) measured by caliper. Data were presented as mean ± SEM, *P < 0.05, **P < 0.01, versus AOM/DSS vehicle group. **C.** *In vivo* tumor suppression effect of Rh2E2 on LLC-1 xenograft via intraperitoneal (IP) injection. **D.** *In vivo* suppressive effect on tumor growth caused by Rh2E2 on LLC-1 xenograft mouse model via oral administration. C57/BL mice were subcutaneously inoculated with 2 × 10^6^ LLC-1 mouse lung cancer cells. The administration of Rh2E2 by either IP or oral feeding was initiated when the tumor volume reached 50 mm^3^. The tumor size and body weight of mice were then monitored and measured daily for consecutive 21 days. **E.** Study on the sub-chronic lethal dose of Rh2E2. C57/BL mice were orally administrated with 160 or 320 mg/kg of Rh2E2 for consecutive 7 days, the survival and body weight of mice were monitored and recorded. **F.** Test of LD50 value of Rh2E2. C57/BL mice were orally administrated with single dose 2000 mg/kg of Rh2E2, the survival rate of mice was monitored and recorded for 14 days. *P < 0.05, **P < 0.01, ***P < 0.001 compared to the vehicle-treated group.

Panax ginseng has been rapidly increasing in the Asian countries and Western world as a dietary supplement for cancer patients. People believe that Panax ginseng is beneficial for humans without observable adverse effects. In this study, no cytotoxicity of Rh2E2 was revealed in both normal and cancer cell cultures. Colorectal cancer (CRC) mouse model was used to investigate the chemopreventive effect of Rh2E2. In this model, tumors are induced by combining a single dose of azoxymethane (AOM) and chronic exposure to the inflammatory agent dextran sodium sulfate (DSS). As shown in Figure 2A, the tumor incidence of the AOM/DSS model was approximately 81%, whereas the administration of Rh2E2 at 20, 40, and 80 mg/kg and the positive control drug aspirin [23] decreased the tumor incidence. The mice treated with Rh2E2 at the dose of 40 mg/kg showed lower tumor number and reduced tumor volume in colon. The effect of Rh2E2 at 40 mg/kg in suppressing the tumor multiplicity and tumor volume is better than aspirin (Figure 2B & Supplementary Figure S1A & S1B). Furthermore, the increase of spleen weight in AOM/DSS mice was attenuated by Rh2E2 (40 and 80 mg/kg) and aspirin (50 mg/kg) (Supplementary Figure S1C). Moreover, the body weight of Rh2E2- and aspirin-treated AOM/DSS mice gradually increased, suggesting no toxic effect of the drugs to animals (Supplementary Figure S1D). Collectively, Rh2E2 possesses a chemopreventive effect against colorectal cancer similar to aspirin.

### Rh2E2 suppresses tumor growth in a lung cancer xenograft mouse model without observable adverse effects

*In vivo* anti-tumor effect of Rh2E2 was further assessed in a lung cancer xenograft model. As shown in Figure 2C, intraperitoneal (IP) injection of Rh2E2 at 5 and 10 mg/kg/day demonstrated dose-dependent inhibition of tumor growth up to 18.72% (*P* < 0.05) and 34.34% (*P* < 0.01), respectively. Treatment with Rh2E2 showed no reduction in body weight or vital organs, suggesting a non-toxic property of Rh2E2 (Supplementary Figures S1E & S2). Oral administration of Rh2E2 at 40 and 80 mg/kg/day demonstrated dose-dependent inhibition of tumor growth up to 44.28% (*P* < 0.05) and 52.2% (*P* < 0.01), respectively, while the positive control drugs 5-Fu, (*R/S*)-Rh2 and Rg3 had little or no anti-tumor effect (Figure 2D). IP injection and oral administration of Rh2E2 neither reduced body weights nor had any observable adverse effects in mice (Supplementary Figures S1E-F, S2 & S3). The therapeutic safety window of Rh2E2 was further evaluated by oral administration of its sub-chronic lethal dose and acute lethal dose. Up to 320 mg/kg/day of Rh2E2 indicated no harmful effect to animals, showing 100% of the animals survived and no decline in body weight after a 7-day treatment course (Figure 2E). The acute oral toxicity of Rh2E2 was assessed using the Organization for Economic Cooperation and Development (OECD) Guideline for Testing Chemicals (the sections “Acute Oral Toxicity” and “Acute Toxic Class Method”). Based on the sub-chronic lethal dose shown in Figure 2E, 2000 mg/kg of Rh2E2 was selected as a starting dose to evaluate the oral acute toxicity of Rh2E2; while death did not occur in animals in either gender, and no decline in body weight and no toxic signs were observed (Figure 2F). According to the OECD Test Guideline 423, Rh2E2 could be either classified as Class 5 or unclassified under the Globally Harmonized Classification System (GHS), and its LD_50_ was therefore estimated to be greater than 5000 mg/kg.

### Rh2E2 inhibits tumor metastasis and induces apoptosis and necrosis in LLC-1 bearing carcinoma of mice

**Figure 3:**
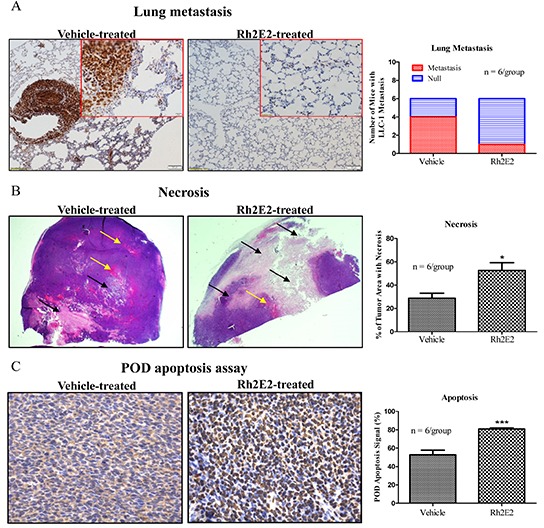
Immunohistochemical analysis of lung tumor tissues from Rh2E2-treated mice **A.** Rh2E2 suppressed the LLC-1 tumor metastasis in lung region of LLC-1 xenograft. Lung tissue sections from Rh2E2 or vehicle control-treated mice were stained with PCNA marker and its signal was visualized by DAB substrate followed by hematoxylin staining. Metastasized LLC-1 cancer cells with strong PCNA signals were visualized in lung tissues and captured from 6 animals of each group. Normal images, 10X magnifications; enlarged images, 40X magnification. Bar chart represented the number of mice with LLC-1 metastasis in lung region. **B.** Rh2E2 enhanced the necrotic areas in tumor tissues of LLC-1 xenograft mice. Tumor sections from Rh2E2 or vehicle control-treated mice were stained with hematoxylin and eosin. The necrotic area was shown in white color (black arrows), blood vessels were indicated as red color (yellow arrows). Bar chart represented the percentage of tumor area with necrosis. **C.** Rh2E2 increased the apoptotic cells in tumor tissue of LLC-1 xenograft. Tumor sections from Rh2E2 or vehicle control-treated mice were analyzed for apoptotic cells using POD kits followed by hematoxylin staining. Bar chart represented the percentage of apoptosis signal in tumor sections of LLC-1 xenograft.

Because tumor metastasis is a leading cause of death in lung cancer patients [24]. Lung tissues from the vehicle- and Rh2E2-treated mice at 10 mg/kg body weight with IP injection were subjected to immunohistochemical (IHC) staining to identify proliferating cell nuclear antigen (PCNA) markers [25]. As shown in Figure 3A, four of the six vehicle-treated mice in the control group gave a strong PCNA signal throughout the lung tissue sections, and the PCNA-stained LLC-1 cancer cells were larger than the normal lung fibroblasts, which suggests that LLC-1 cancer cells metastasized from the subcutaneous dorsal region to the lung tissues. Only one of the six Rh2E2-treated mice had PCNA-stained LLC-1 cells in the lung tissues, which indicated that Rh2E2 could inhibit the metastasis of LLC-1 cancer cells *in vivo* (Figure 3A). The average percentage of tumor necrotic areas (black arrow) reached approximately 30% in vehicle-treated mice, in which tumor tissues were accompanied with rich blood vessels (yellow arrow), whereas the average percentage of tumor necrotic areas were increased, up to approximately 60%, in Rh2E2-treated animals, in which the tumor tissues contained less blood vessel formation (Figure 3B). *In Situ* cell death detection assay (POD) demonstrated that Rh2E2 enhanced apoptotic signaling compared to vehicle-treated mice (Figure 3C). Thus, Rh2E2 could suppress tumor growth via the induction of necrosis and apoptosis.

### Rh2E2 down-/up-regulates protein expression involving invasion, proliferation, cell cycle progression and apoptosis of cancer cells

We have optimized the method of isoelectric focusing (IEF) in 2-dimensional gel electrophoresis (2D) of mouse tumor tissues, leading to an enhancement of protein identification and reproducibility [26, 27]. We therefore combined 2D-DIGE and iTRAQ proteomic techniques to maximize the identification of protein spots from tumor tissues of Rh2E2-treated mice. Among 2670 protein spots identified in 2D-DIGE-MALDI-TOF/TOF using peptide mass fingerprinting, 48 proteins in tumor tissues (*p* < 0.05) were differentially expressed between the vehicle- or Rh2E2-treated animals; 34 proteins were down-regulated and 14 proteins were up-regulated (Supplementary Table S1). A total of 6667 protein spots were identified by using iTRAQ analysis. Among these identified proteins, 98 proteins (*p* < 0.05) were differentially expressed between the vehicle- and Rh2E2-treated mice. In Rh2E2-treated mice, 59 proteins were up-regulated, whereas 39 proteins were down-regulated (Supplementary Table S2).

**Figure 4:**
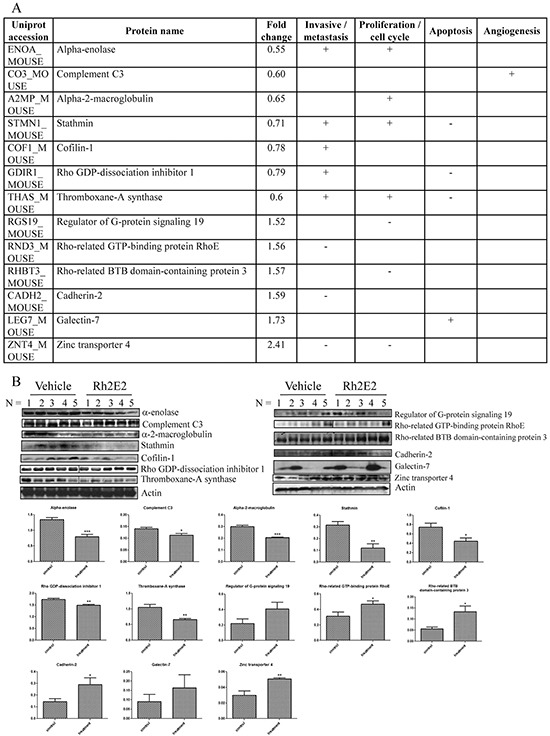
Proteomic analysis on the tumor tissues from Rh2E2- or vehicle-treated LLC-1 xenograft mice **A.** Rh2E2-induced differential expression of proteins were associated with invasive/metastasis, cell proliferation, apoptosis and angiogenesis of cancer cells. The comparative proteomic analyses, iTRAQ-LC-MS/MS and 2D-DIGE, were combined to maximize the identification of differentially expressed proteins. For iTRAQ, the peptide-IEF was used for fractionation before LC-MS/MS. MS/MS data was processed using Bruker Compass Data Analysis software, and the generated peak lists were submitted to MASCOT search engine against SwissProt 51.6 database. For 2D-DIGE, gels were scanned using a Typhoon 9400 (GE Healthcare) laser scanner. Image analysis was carried out with DeCyder differential analysis software 7.0 (GE Healthcare). The differentially expressed protein spots were identified by MALDI TOF/TOF. The MS data were analyzed using FlexAnalysis 3.3 (Bruker Daltonics) and the generated peak list was searched against SwissProt Mus musculus protein database (SwissProt 57.1, 462764 sequences; 163773385 residues) using in-house MASCOT Server software, version 2.3 (Matrix Science, London, UK). **B.** Immunoblotting validation of the identified protein targets. The protein bands intensity for corresponding protein was quantified using image J software. All quantitative data were given as mean ± SEM. Statistical analyses were performed using the non-paired Student's *t*-test (GraphPadPrism, GraphPad Software Inc., San Diego, CA, US) to compare means. *P < 0.05, **P < 0.01.

In a literature review of these 146 differentially expressed proteins identified by 2D-DIGE and iTRAQ, 13 proteins were associated with cancer cell invasion and metastasis, cell proliferation and cycle progression, apoptosis and angiogenesis [28–39] (Figure 4A). Among those 13 proteins, 6 were identified by 2D-DIGE analysis, including α-enolase, complement C3, alpha-2-macroglobulin, stathmin, cofilin-1 and Rho GDP-dissociation inhibitor 1 (Supplementary Figure S4), whereas 7 were identified from iTRAQ analysis, including thromboxane-A synthase, regulator of G-protein signaling 19, Rho-related GTP-binding protein RhoE, Rho-related BTB domain-containing protein 3, cadherin-2, zinc transporter 4, and galectin-7 (Figure 4A). These results suggested that the combination of the 2D-DIGE and iTRAQ analytical methods could enhance the coverage of protein identification. Furthermore, the identified proteins were validated by Western blotting (Figure 4B). Taken together, Rh2E2 could suppress *in vivo* tumor growth via modulation of proteins involved in cell invasion, proliferation, cell cycle progression and apoptosis of cancer cells.

### Knockdown of α-enolase and stathmin in H1299 potentiates the Rh2E2-inhibited cancer cell invasion

**Figure 5:**
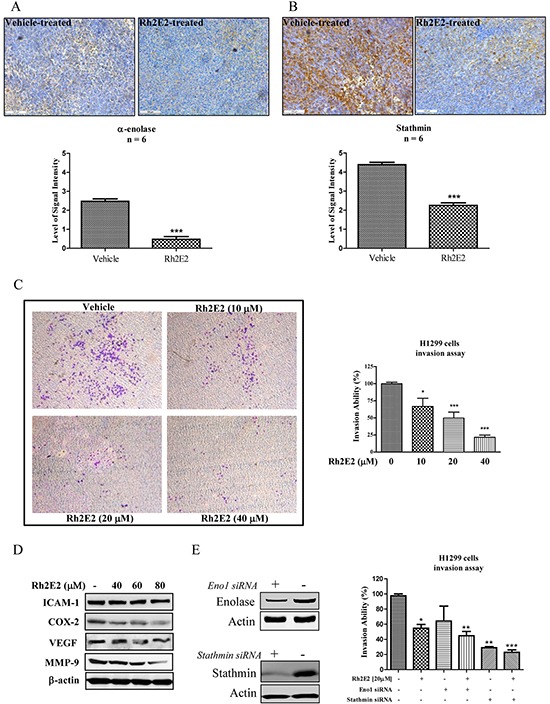
The role of α-enolase and stathmin in Rh2E2-inhibited cancer cells invasion **A.** Rh2E2 suppressed expression of α-enolase in tumor tissues of Rh2E2-treated LLC-1 xenograft mice. **B.** Rh2E2 suppressed the expression of stathmin in tumor tissues of Rh2E2-treated LLC-1 xenograft mice. α-enolase and stathmin staining images were representative of 5 tumor sections from 6 animals of each group. The level of signal intensity was scored from 1-5 (5 is maximum) and took average from five different views of each section taken in 20X magnifications. **C.** Rh2E2 dose-dependently inhibited the cell invasion ability of H1299 lung cancer cells. Images of invasive cells found in the lower layer of ECMatrixTM chamber were captured by Digital Camera under microscope with 40X magnifications. Bar chart represented the percentage of invasion ability of the stained invasive cells solute. **D.** Rh2E2 suppressed the expression markers for cell invasion and angiogenesis including ICAM-1, COX-2, VEGF and MMP-9. **E.** siRNA knockdown of α-enolase and stathmin potentiated the anti-invasive effect of Rh2E2. H1299 cancer cells were transfected with control or α-enolase and stathmin siRNA for 48 hours, the knockdown cells were then subjected to cell invasion assay using ECMatrix™ chamber followed by the treatment of Rh2E2. *P < 0.05, **P < 0.01 and ***P < 0.001 compared to medium control.

In view of the anti-metastatic effect of Rh2E2 *in vivo* (Figure 3A), proteomic profiling provided evidence that several tumor metastatic markers, such as α-enolase, stathmin, cofilin-1, Rho GDP-dissociation inhibitor 1 and thromboxane-A synthase [31–34, 40], probably participate in the anti-metastatic actions of Rh2E2. The suppressed metastatic proteins, α-enolase and stathmin, were further studied. Consistent with the Western blot validation shown in Figure 4B, IHC staining of tumor tissues confirmed that both α-enolase and stathmin were suppressed in Rh2E2-treated mice (Figure 5A & 5B). Transwell chamber assays showed that sub-lethal doses of Rh2E2 could dose-dependently suppress the cell invasion ability of the human lung cancer cells H1299 (Figure 5C). Protein markers for cell adhesion, cell invasion and angiogenesis were all down-regulated upon Rh2E2 treatment (Figure 5D). Knockdown of α-enolase and stathmin in H1299 cells retarded the cell's invasive ability, and Rh2E2 in these knockdown cells further destroyed the invasive ability of H1299 (Figure 5E). The suppression of either α-enolase or stathmin is required for the Rh2E2-retarded cancer cell invasion and tumor metastasis.

### Rh2E2 specifically suppresses cancer cell metabolism via inhibition of metabolic enzymes in mitochondrion

**Figure 6:**
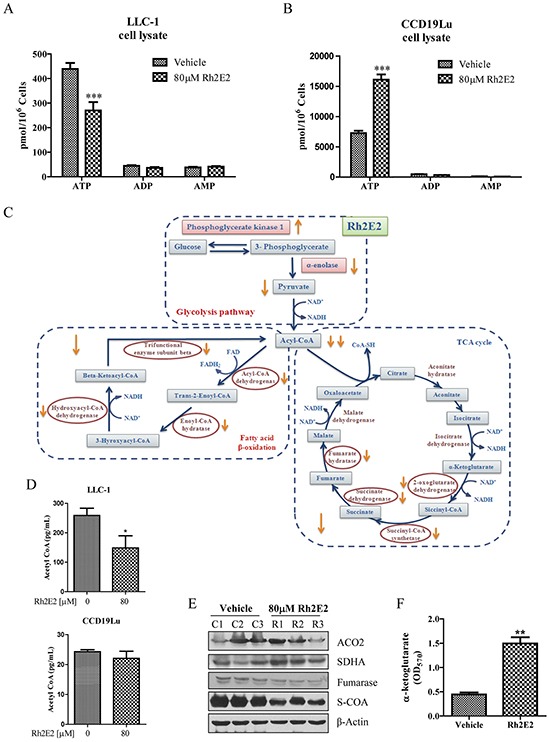
Effect of Rh2E2 on the network-based metabolic reprograming of LLC-1 cancer cells **A.** Rh2E2 decreased the ATP production in LLC-1 cancer cells. **B.** Rh2E2 enhanced ATP generation in CCD19Lu normal cells. Equal numbers of medium control or Rh2E2-treated cells (LLC-1 or CCD19Lu) were harvested for ATP, ADP and AMP determination by UPLC-MS quantitation with their reference control. The amount of energy metabolites was calculated as pmol/10^6^ cells. **C.** Schematic diagram of network-based metabolic intervention of Rh2E2 in LLC-1 lung cancer cells. Mitochondrial proteins from Rh2E2-treated LLC-1 cancer cells or CCD19Lu normal cells were harvested for proteomic analysis using iTRAQ. Three biological replicates were compared and analyzed to reduce the variation caused by random biological effects. **D.** Rh2E2 specifically reduced the production of acetyl CoA in LLC-1 lung cancer cells. **E.** Immunoblotting validation of the identified metabolic enzymes in TCA cycle. **F.** Rh2E2 enhanced the accumulation of α-ketoglutarate, the energy metabolite of TCA cycle.

α-enolase is one of the major glycolytic enzyme used in ATP energy production [28]. Tumor cells require a large increase in glucose metabolism to support tumor formation and expansion by inducing the overexpression of glycolytic enzymes [28]. We therefore determined whether the down-regulation of α-enolase by Rh2E2 could decrease glycolysis and energy production. As shown in Figure 6A and 6B, an equal number of LLC-1 cancer cells generated less ATP than that of the normal cells did, owing to the Warburg effect [2], because the cancer cells adopted the glycolytic pathway, which yields less ATP than the mitochondrial aerobic oxidation utilized by normal cells. Rh2E2-treated LLC-1 cancer cells showed a decrease in ATP production without affecting the ADP and AMP levels (Figure 6A). By contrast, Rh2E2-treated human normal lung fibroblasts could effectively reverse the level of ATP production (Figure 6B), which suggests that Rh2E2 exhibits a biphasic effect on energy production between normal and cancer cells. We addressed whether Rh2E2 treatment suppresses the key metabolic enzymes of mitochondria. As shown in Figure 6C, proteomic analysis revealed that several key enzymes involved in fatty acid β-oxidation, including acyl-CoA dehydrogenase, enoyl-CoA hydratase, 3-hydroxyacyl-CoA dehydrogenase and beta-ketoacyl-CoA thiolase were all down-regulated in Rh2E2-treated LLC-1 cancer cells but not in normal lung fibroblasts (Supplementary Tables S3 & S4). Concomitantly, the level of metabolic enzymes in the TCA cycle including aconitase, α-ketoglutarate dehydrogenase, succinyl-CoA synthetase, succinic dehydrogenase and fumarase were decreased in Rh2E2-treated LLC-1 cancer cells (Supplementary Table S3). We further validated the down-regulation of metabolic enzymes in fatty acid β-oxidation by examining their end product, acetyl-CoA. In line with the proteomic findings, a reduction in acetyl-CoA was observed in Rh2E2-treated LLC-1 cells, but not in Rh2E2-treated CCD19Lu normal lung fibroblasts, which suggests that neither the metabolic enzymes nor the end product of fatty acid β-oxidation were affected in normal cells by Rh2E2 (Figure 6D). Western blot analysis confirmed that aconitase was up-regulated, whereas succinyl-CoA synthetase and fumarase were down-regulated in Rh2E2-treated LLC-1 cells (Figure 6E). Up-regulation of aconitase together with down-regulation of α-ketoglutarate dehydrogenase led to the accumulation of the TCA cycle intermediate, α-ketoglutarate. Rh2E2 increased-α-ketoglutarate in LLC-1 cells (Figure 6F), indicating the TCA cycle was retarded upon Rh2E2 treatment. These findings suggest that Rh2E2 specifically suppress cell metabolism and energy production in cancer cells, but not in normal cells.

### Rh2E2 induces S-phase cell cycle arrest in LLC-1 cancer cells through decreasing S-phase specific cyclin-dependent kinases (cdks)/cyclins expression

Metabolic disturbance could influence cell proliferation and cell cycle progression [41]. Results indicated that Rh2E2 arrested the cells in S-phase of LLC-1 cancer cells (Supplementary Figure S5A). The percentage of cells in the S-phase was 56.46 ± 3.60% in Rh2E2-treated LLC-1 cancer cells compared to DMSO-treated cells (28.72 ± 1.88%) (*P* < 0.001). Rh2E2 treatment did not affect cell cycle progression in the normal lung fibroblasts CCD19Lu (Supplementary Figure S5B), suggesting that Rh2E2 could specifically inhibit the proliferation of LLC-1 cancer cells by inducing S-phase cell cycle arrest. These findings were in agreement with the results of proteomic analysis, especially the reduction of energy metabolism.

We then determined the regulators responsible for S-phase cell cycle arrest upon Rh2E2 treatment. As shown in Supplementary Figure S5C, Rh2E2 decreased the expression of cyclin D1 and cdk4, with a moderate decrease in cyclin A, while the expression of cyclin E and cdk2 was reduced. These results are in agreement with other reports that inhibition of the cdk/cyclin complex activity suppresses cell cycle progression [42]. p21 is known to bind and inhibit the activity of the cdk4/cyclin D1 complex, whereas p27 could suppress the activity of the cdk2/cyclin E complex [43]. Recent studies have indicated that the tumor suppressor proteins p21 and p27, as well as p53, suppress the AMPK-related metabolic pathway and glucose metabolism [44, 45]. We therefore postulated that Rh2E2 may down-regulate cancer cell metabolism as well as arrest cell cycle progression via activation of these tumor suppressors. As expected, Rh2E2 caused the accumulation of both p21 and p27 in a dose-dependent manner with the activation of p53. The expression of c-myc, which acts as a transcriptional factor for cdks, was also decreased (Supplementary Figure S5D). Immunoprecipitation (IP) assay showed that the level of cyclin E or cyclin D in both cdk2/cyclin E and cdk4/cyclin D complexes was reduced in response to Rh2E2 treatment (Supplementary Figure S5E). IP assay with p21 or p27 further revealed that the level of cdk2 or cdk4 was increased in a dose-dependent manner (Supplementary Figure S5F), suggesting an enhanced binding inhibition between both p21 and p27 with cdk/cyclin complexes upon Rh2E2 treatment. Upon knockdown of p21 and p27 in LLC-1 cells (Supplementary Figure S6A), the cell viability could be partially recovered from cell death induced by Rh2E2 (Supplementary Figure S6B). Concomitantly, Rh2E2-induced S-phase cell cycle arrest was partially attenuated in LLC-1 cells after knockdown of p21 or p27 (Supplementary Figure S6C). These findings provide evidence that Rh2E2 could enhance the expression of p21 and p27, which would bind to cdks and cyclins and suppress their interaction, leading to a loss of cdks/cyclins complex activity, and contributing to S-phase cell cycle arrest and cell cytotoxicity.

### Expression of p27 in Rh2E2-treated LLC-1 cancer cells relies on inhibition of the Skp2 autoinduction loop

**Figure 7:**
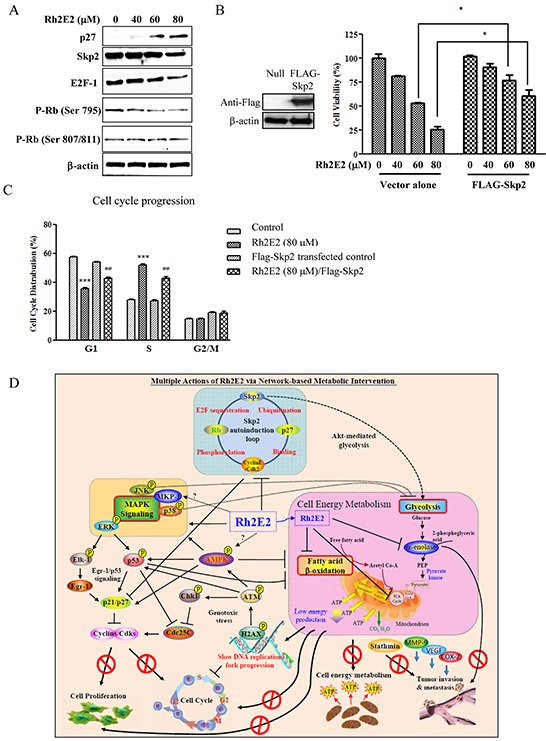
The role of Skp2 in Rh2E2-induced anti-cancer effect **A.** Effect of Rh2E2 on Skp2 signaling pathway. **B.** Overexpression of Skp2 reduced cytotoxic effect of Rh2E2. **C.** Overexpression of Skp2 diminished the S-phase cell cycle arrest of Rh2E2. (*t*-test: ***P < 0.001 for Rh2E2 comparing with non-transfected group; ##*P* < 0.01 for Skp2 transfected cells with Rh2E2 treatment comparing with non-Skp2 transfected Rh2E2 treatment group). **D.** Multi-dimensional actions of Rh2E2 via energy-based metabolic intervention.

Skp2 is a substrate-targeting protein subunit of the SCFskp2 ubiquitin ligase complex that controls cell proliferation, especially promoting entry into and progression through the S phase, by regulating the degradation of p27 [46]. A recent report introduced the concept of a Skp2 autoinduction loop, which is a self-amplifying feedback loop compromising Skp2, p27, Cyclin E/Cdk2 and the retinoblastoma tumor suppressor gene Rb [47]. In 2013, Chan *et al.* further unraveled the role of Skp2 in Akt-induced glycolysis and p53-independent cellular senescence [48]. We hypothesized that Rh2E2 may interrupt cancer cell energy production and cell cycle progression via the Skp2 autoinduction loop. As shown in Figure 7A, an increase in p27 was accompanied by a reduction of skp2 and E2F-1 expression, as well as dephosphorylation of Rb. We further addressed the role of skp2 in Rh2E2-induced cell cytotoxicity and cell cycle arrest by overexpression of FLAG-tagged skp2 in LLC-1 cells. Overexpression of skp2 could enhance cancer cell viability in the presence of Rh2E2 (Figure 7B), suggesting that Rh2E2-enhanced cytotoxicity was offset by skp2. Rh2E2 in skp2 overexpressing LLC-1 cells contributed to S-phase cell cycle arrest, although to a lesser extent (Figure 7C). Taken together, Rh2E2 arrests LLC-1 cancer cell growth in the S-phase by up-regulation of p27 via abating the skp2 autoinduction loop.

### Rh2E2-enhanced cell cytotoxicity requires activation of ERK-p53 and ATM-AMPK signaling

Recent reports indicate that MAPK signaling contributes to the maintenance of glucose homeostasis and peripheral tissue energy balance [49]. We found that ERK activation was required for Rh2E2-induced cytotoxicity (Supplementary Figure S7A, B); while co-treatment of Rh2E2 with U0126 resulted in a decrease in both ERK- and p53-activated phosphorylation as well as alleviation of p21 and p27 activation (Supplementary Figure S7C). These results suggest that ERK is involved in Rh2E2-reduced cancer cell metabolism and cell death through an ERK/Egr-1/p21 or /p27 and an ERK/p53/p21 or /p27 -dependent pathway. By contrast, recent studies revealed that bioenergetic metabolic reduction correlates with slower DNA replication fork progression and ensures cell cycle arrest, leading to senescence [50]. Apparently, Rh2E2 reduced DNA synthesis in LLC-1 cancer cells in a dose-dependent manner (Supplementary Figure S8A). H2A.X is activated in the retention of the DNA repair factory at DNA damage sites; it is directly phosphorylated by ATM in response to DNA damage [51]. Our results indicated that H2A.X was phosphorylated upon Rh2E2 treatment in LLC-1 cancer cells, but not in CCD19Lu normal cells (Supplementary Figure S8B). These results were in agreement with our data showing that Rh2E2 induced specific cytotoxicity, S phase cell cycle arrest and cell metabolic modulation in LLC-1 cancer cells. Phosphoinositide 3-kinase-related kinases (ATM and ATR) are activated in response to DNA damage/genotoxic stress for early signal transmission through the cell cycle checkpoints [52]. We demonstrated that Rh2E2 could increase ATM protein expression and AMPK and Chk1 phosphorylation, but had little effect on ATR protein expression, whereas ultra-violet (UV) radiation, a well-known activator of ATM/ATR, induced a high expression of both kinases (Supplementary Figure S8C & S8D). By contrast, blockage of Rh2E2-activated ATM/ATR signaling using specific inhibitors was able to reduce the Rh2E2-induced cell death and S phase cell cycle arrest (Supplementary Figure S8E & S8F). These results suggested the possible involvement of ATM-AMPK signaling in Rh2E2-induced cell metabolic suppression and S phase cell cycle arrest.

## DISCUSSION

Small-molecule agents for the specific suppression of tumors that spare normal host tissues have received increasing attention in recent years [53]. In the past two decades, chemicals derived from ginsenosides or active components from ginseng have been tested [54], providing solid data to support the application of ginseng in cancer therapy. The cell energy sensor AMPK has recently been reviewed as a target for ginseng [55], which suggests that ginseng or ginsenosides would be valuable in the treatment and/or prevention of cancer via inhibition of energy metabolism.

In the current study, we synthesized a ginsenoside derivative, Rh2E2, based on structural modification of 20(*R*)-Rh2 that has no anti-cancer effect. In addition to the tumor specific properties of Rh2E2 *in vitro*, it demonstrated a potent chemopreventive effect in an AOM/DSS colon cancer mouse model with a potency comparable to aspirin, and effectively suppressed tumor growth and metastasis in a LLC-1 mouse xenograft by oral and IP administration. The acute and sub-chronic lethal doses of Rh2E2 could not be determined, even when the dosages of Rh2E2 reached a maximum oral dose, because of the very low toxicity of the drug, which suggests that the range of the safe therapeutic window is large. Rh2E2 was found to reduce the glycolytic enzyme α-enolase and the metabolic enzymes involving fatty acid β-oxidation, leading to a reduction in ATP production. As a result, the LLC-1 cancer cells with reduced metabolism were arrested at the S-phase of the cell cycle via activation of the Skp2 autoinduction loop, induction of ERK-p53 signaling, suppression of cdk/cyclin complexes via cdk inhibitors of p21 and p27, and induction of genotoxic stress (Figure 7D).

The enzyme α-enolase is a diagnostic and therapeutic target of cancers [56]. It is a glycolytic enzyme in the synthesis of pyruvate and also a multifunctional protein that acts as a plasminogen receptor to promote cell migration and metastasis [57]. In cancer cells, α-enolase is overexpressed, thus enhancing anaerobic proliferation (Warburg effect) [58]. Also. it is localized at the cell surface where it facilitates cancer cell invasion [56]. The current results showed that Rh2E2 had no influence on energy metabolism in normal cells and tissues. Instead, it had suppressive effect on carcinoma growth by inhibition of energy-based metabolism through suppression of α-enolase, leading to a selective reduction of ATP energy production, and inhibition of tumor cell invasion and metastasis *in vivo*. Extracellular acidification has been shown to increase the motility of cells both *in vitro* and *in vivo*, and lactate itself has been shown to directly increase cell motility [59]. Rh2E2 could suppress glycolysis via the suppression of α-enolase, leading to blockage of pyruvate and lactate production. Together, Rh2E2 is able to intervene in tumor acidification and inhibit tumor cell invasion.

Recent studies found that cancer cells lacking the brain-specific metabolic enzyme carnitine palmitoyltransferase 1C (CPT1C) showed decreased fatty acid oxidation, reduced ATP generation and increased sensitivity to metabolic stress [60]. Pharmacological inhibition of fatty acid oxidation could sensitize human leukemia cells to cell death [61]. These observations suggest that inhibition of fatty acid oxidation may be a therapeutic mechanism in treating cancers. Rh2E2 not only down-regulated the key metabolic enzymes for fatty acid oxidation and led to a reduction of acetyl-CoA (a key substrate of the TCA cycle) in LLC-1 cancer cells, but also reduced the expression of some mitochondrial enzymes of the TCA cycle, producing an accumulation of α-ketoglutarate. Recent studies demonstrated that glutamate dehydrogenase 1 controls the intracellular level of α-ketoglutarate, which is important for maintaining redox homeostasis in cancer cells. Dysregulation of this mitochondrial enzyme would lead to an accumulation of α-ketoglutarate and imbalanced redox homeostasis, leading to attenuated cancer cell proliferation and tumor growth [62]. Our findings suggest that Rh2E2 suppressed tumor cell growth via multiple metabolic interventions, such as energy-based metabolic suppression, by inhibiting glycolysis, fatty acid oxidation and the TCA cycle for ATP generation.

The tumor suppressor p53 can shut down aerobic glycolysis (Warburg effect) in cancer cells and also renders cells to utilize oxidative phosphorylation in mitochondria, thereby minimizing the synthesis of substrates for cell division [63]. Skp2 promotes cell proliferation and cell cycle progression in the S-phase by down-regulating the cdk inhibitor p27 [46]. Apparently, Rh2E2 decreased energy production by suppressing aerobic glycolysis in cancer cells, and on the other hand, it arrested the cell cycle in S-phase with up-regulation of p53 and p27 and down-regulation of Skp2. These results suggest that the energy-based actions of Rh2E2 facilitate crosstalk among those signaling pathways. Studies showed that Skp2 deficiency impairs Akt activation, glucose transporter 1 expression, and glucose uptake and glycolysis, and suppresses cancer progression in various tumor models [64]. Recently, a newly identified Skp2 inhibitor suppresses Akt-mediated glycolysis as well as triggering p53-independent cellular senescence [48]. These findings provide evidence that Rh2E2 reduces cancer cell metabolism via crosstalk between Skp2 and p53 signaling, thereby arresting cancer cell growth in the S-phase. Skp2 inhibitor has anti-cancer effect in various animal models and cooperates with chemotherapeutic agents to reduce cancer cell survival [48]. Those reports not only provide evidence that Skp2 is a target for restricting cancer cell progression, but also suggest that Rh2E2 is a potent anti-cancer agent by suppressing Skp2.

It is inevitable that Rh2E2-suppressed energy metabolism in cancer cells would eventually inhibit nucleic acid synthesis and induce genotoxic stress, because DNA synthesis is an energy-demanding process [65]. The anti-tumor effect of Rh2E2 was demonstrated by the induction of genotoxic stress on LLC-1 cancer cells, but not in normal lung fibroblasts through activation of Chk1 and ATM signaling, leading to phosphorylation of the genotoxic stress marker H2A.X, and a reduction in DNA synthesis. However, induced genotoxic stress may be an indirect mechanism for the anti-tumor effect of Rh2E2 due to its energy-based inhibition of cellular metabolism in cancer cells. In normal proliferating cells, the exogenous pool of nutrients, such as nucleosides, can supply the nucleotide building blocks. However, because of the high proliferative burden of cancer cells, tumors probably rely on endogenous synthesis from glucose and glutamine via the pentose phosphate pathways (PPP), glutaminolysis and the tricarboxylic acid cycle (TCA cycle) [66]. Therefore, the down-regulation of metabolic enzymes involving glycolysis, fatty acid β-oxidation and the TCA cycle induced by Rh2E2, might indirectly block nucleotide biosynthesis and induce genotoxic stress in cancer cells. Rh2E2 exhibits no suppressive effect on the above metabolic pathways in normal lung fibroblasts, showing no genotoxic stress markers in normal cells. Recently, activation of ATM signaling has been shown to suppress energy metabolism via activation of the AMP-activated protein kinase, AMPK, which is an energy sensor of cells [67]. ATM appears to phosphorylate other signal transducers with metabolic connections, including p53 [67], suggesting that Rh2E2 might further suppress cell metabolism via ATM-AMPK-p53 signaling.

The metabolic therapeutic agent metformin has a potent anti-tumor effect on melanoma, lung, breast, lymphoma, liver, colorectal, prostate, pancreatic, gastric, ovarian and cervical cancers, either in pre-clinical studies or in clinical trials, through a multi-pathway intervention in cancer metabolism [68, 69]. The effective dosages of metformin for tumor suppression in mice are approximately 50 mg/day for oral administration [70] and 200mg/kg/day for IP injection [71]; those dosages are much higher than the effective dosage of Rh2E2 (1 mg/day for oral administration and 10 mg/kg/day for IP injection). Most importantly, Rh2E2 had no observable side effects at effective dosages in mouse models, and has an LD_50_ value of 5000 mg/kg, whereas metformin could induce lactic acidosis, which may be a considerable safety issue in clinical applications [17]. The effective dosage of Rh2E2 is higher than that of other chemotherapeutic agents such as paclitaxel as a first-line anti-cancer agent, but Rh2E2 may be more suitable for developing combined therapy with other first-line chemotherapeutic agents to produce synergistic therapeutic efficacy for cancers. The glycolysis inhibitor, 2-deoxyglucose can resensitize tumors to paclitaxel through reducing tumor ATP levels [12]. Down-regulation of α-enolase can further enhance the sensitivity of tumor cells to anti-tubulin chemotherapeutics (e.g., vincristine and taxol) [72]. Therefore, the pharmacological property of Rh2E2 of specific cytotoxicity to cancer cells but not to normal cells, as well as the wide range of the therapeutic safety window, suggests that it could be developed as a safe and effective adjuvant agent in treating cancer together with current chemotherapeutic agents.

## MATERIALS AND METHODS

### Preparation of ginsenoside Rh2E2

A mixture of Oxone® mono-persulfate compound (494.2 mg) and NaHCO_3_ (210.4 mg) was added slowly to a solution of 20*R*-ginsenoside Rh2 (100 mg) in 60 mL of a 1:1 mixture of acetonitrile-Na_2_(EDTA) (4 × 10^−4^ M in water). Shi epoxidation diketal catalyst (Ketone, 124.5 mg) in 15 mL of acetronitrile was then added dropwise during a period of 10 minutes. The reaction mixture was allowed to stand overnight at room temperature with magnetical stirring. After filtration and removal of acetronitrile in vacuum, the reaction solution was directly loaded to an ODS column and eluted from 50% to 90% methanol to afford 20*R*-Rh2 20,24-epoxides (70 mg) in an equivalent mixture of 24-epimers, *i.e.* 3-O-*β*-D-glucopyranosyl 20*R*,24*S*-epoxydammarane-3*β*,12*β*-triol and 3-O-*β*-D-glucopyranosyl 20*R*,24*R*-epoxydammarane-3*β*,12*β*-triol (Rh2E2) as illustrated in Scheme 1. Identification of Rh2E2 was described in supplementary information. Several grams of Rh2E2 were synthesized according to the above procedure.

### Cell culture, cytotoxicity assay, apoptosis detection, *siRNA* knockdown, cell cycle analysis and cell invasion assay

All cells were obtained from the American Type Culture Collection (Rockville, MD) unless otherwise specified. Cells were characterized by ATCC according to their guidelines on cell line verification test recommendations. 20(*S*)-Rh2 and 20(*R*)-Rh2 were used as references control drugs. Rh2E2 or two reference compounds were freshly prepared from 100 μM stock solution in DMSO. Cell viability, Western blot detection and immunofluorescence staining were measured and monitored as described previously [73]. Apoptosis was detected by Annexin V staining kit (BD Biosciences). *siRNA* knockdown was performed using X-tremeGENE siRNA Transfection Reagent (Roche). To investigate the effect of MAPK inhibitors on Rh2E2-induced cell death, confluent cell cultures were co-incubated with Rh2E2 in the presence of the following inhibitors: 20 μM U0126 (ERK inhibitor), 2.5 μM SP600125 (JNK inhibitor), 2.5 μM SB203580 (p38 inhibitor), 10 μM Pifithrin-α (p53 inhibitor). For cell cycle analysis, the cells were harvested and washed with ice-cold phosphate-buffered saline (PBS), and then suspended and permeabilized with 70% ethanol for overnight at 4°C. For detecting DNA content and cell cycle, cells were incubated with the freshly prepared propidium iodide (PI) staining buffer for 30 min at room temperature in dark. Fractions of the cells in G1, S, and G2/M phase were analyzed using Modfit software 3.1. The cancer cell invasion assay was performed in a Cell Invasion Chamber, a 24-well tissue culture plate with cell culture inserts that contain an 8 mm pore size polycarbonate membrane over a thin layer of dried ECMatrix™ (CHEMICON). Detail protocol was described in supplementary information.

### AOM/DSS colorectal cancer model

Male Balb/c mice (4-week-old) from Charles River Laboratory (Horsham, PA, USA) were maintained on AIN-93M diet (Research diet, NJ, USA) and kept in an air-conditioned room with controlled temperature, humidity, and 12h day/night cycle. An azoxymethane (AOM)/dextran sodium sulfate (DSS)-induced colitis-associated colon carcinogenesis model was adopted to evaluate the chemopreventive of Rh2E2. Detail methodologies were described in supplementary information.

### LLC-1 Xenograft model and immunohistochemistry

LLC-1 tumor inoculation, drug treatment and tumor measurement procedure, and IHC staining were described previously [25]. Detail methodologies were described in supplementary information.

### Proteomic analysis of LLC-1 tumor tissues and cells

50 mg of frozen mouse tumor tissues were extracted using TissueLyser LT (QIAGEN, Hilden, Germany) with urea/thiourea lysis buffer (1:10 w/v). For cell lines, the cell pellets were extracted with urea/thiourea lysis buffer [7 M urea, 2 M thiourea, 4 % (w/v) CHAPS, 30 mM Tris/HCl and protease inhibitor, pH 9.0, (GE healthcare)]. The supernatants were processed with 2-D Clean Up kit and re-suspended in the urea/thiourea lysis buffer for 2D-DIGE or in Dissolution buffer containing 5 % SDS provided in iTRAQ Reagent 4-Plex kit (AB SCIEX) for iTRAQ experiment as previously described [26, 27]. MS/MS data was processed using Bruker Compass Data Analysis software, and the generated peaklists were submitted to MASCOT search engine against SwissProt 51.6 database. Detail methodologies were described in supplementary information.

### LC-MS/MS measurement of ATP metabolites

Quantification of energy metabolites was performed using multiple reaction monitoring (MRM) as previously described [74]. Detail methodologies were described in supplementary information.

### Acetyl-CoA assay and α-Ketoglutarate assay

LLC-1 cells were treated with 80 μM Rh2E2 for 24 h. The cell lysates were then harvested for determination of acetyl-coenzyme A (Acetyl-CoA) and α-Ketoglutarate (α-KG) by Acetyl-CoA Assay Kit and α-KG Assay Kit (Sigma, MO, USA) following manufacturer's instruction. Detail methodologies were described in supplementary information.

### Statistical analysis

Results were expressed as means ± S.E.M. as indicated. The difference was considered statistically significant when the *P*-value was less than 0.05 using Prism 5.0 software. Student's *t-test* or one-way *ANOVA* analysis was used for comparison among different groups.

## SUPPLEMENTARY EXPERIMENTAL PROCEDURES, FIGURES AND TABLES



## References

[1] Groves AM, Win T, Haim SB, Ell PJ (2007). Non-[18F]FDG PET in clinical oncology. Lancet Oncol.

[2] Gatenby RA, Gillies RJ (2004). Why do cancers have high aerobic glycolysis?. Nat Rev Cancer.

[3] Vander Heiden MG (2011). Targeting cancer metabolism: a therapeutic window opens. Nat Rev Drug Discov.

[4] Tennant DA, Duran RV, Gottlieb E (2010). Targeting metabolic transformation for cancer therapy. Nat Rev Cancer.

[5] Tennant DA, Duran RV, Boulahbel H, Gottlieb E (2009). Metabolic transformation in cancer. Carcinogenesis.

[6] Semenza GL, Jiang BH, Leung SW, Passantino R, Concordet JP, Maire P, Giallongo A (1996). Hypoxia response elements in the aldolase A, enolase 1, and lactate dehydrogenase A gene promoters contain essential binding sites for hypoxia-inducible factor 1. J Biol Chem.

[7] Pizer ES, Lax SF, Kuhajda FP, Pasternack GR, Kurman RJ (1998). Fatty acid synthase expression in endometrial carcinoma: correlation with cell proliferation and hormone receptors. Cancer.

[8] Parsons DW, Jones S, Zhang X, Lin JC, Leary RJ, Angenendt P, Mankoo P, Carter H, Siu IM, Gallia GL, Olivi A, McLendon R, Rasheed BA (2008). An integrated genomic analysis of human glioblastoma multiforme. Science.

[9] Yan H, Parsons DW, Jin G, McLendon R, Rasheed BA, Yuan W, Kos I, Batinic-Haberle I, Jones S, Riggins GJ, Friedman H, Friedman A, Reardon D (2009). IDH1 and IDH2 mutations in gliomas. N Engl J Med.

[10] Kim JW, Tchernyshyov I, Semenza GL, Dang CV (2006). HIF-1-mediated expression of pyruvate dehydrogenase kinase: a metabolic switch required for cellular adaptation to hypoxia. Cell Metab.

[11] Christofk HR, Vander Heiden MG, Harris MH, Ramanathan A, Gerszten RE, Wei R, Fleming MD, Schreiber SL, Cantley LC (2008). The M2 splice isoform of pyruvate kinase is important for cancer metabolism and tumour growth. Nature.

[12] Maschek G, Savaraj N, Priebe W, Braunschweiger P, Hamilton K, Tidmarsh GF, De Young LR, Lampidis TJ (2004). 2-deoxy-D-glucose increases the efficacy of adriamycin and paclitaxel in human osteosarcoma and non-small cell lung cancers in vivo. Cancer Res.

[13] Leung EL, Cao ZW, Jiang ZH, Zhou H, Liu L (2013). Network-based drug discovery by integrating systems biology and computational technologies. Brief Bioinform.

[14] Rena G, Pearson ER, Sakamoto K (2013). Molecular mechanism of action of metformin: old or new insights?. Diabetologia.

[15] Collier CA, Bruce CR, Smith AC, Lopaschuk G, Dyck DJ (2006). Metformin counters the insulin-induced suppression of fatty acid oxidation and stimulation of triacylglycerol storage in rodent skeletal muscle. Am J Physiol Endocrinol Metab.

[16] Kirpichnikov D, McFarlane SI, Sowers JR (2002). Metformin: an update. Ann Intern Med.

[17] Stang M, Wysowski DK, Butler-Jones D (1999). Incidence of lactic acidosis in metformin users. Diabetes Care.

[18] Oh M, Choi YH, Choi S, Chung H, Kim K, Kim SI, Kim DK, Kim ND (1999). Anti-proliferating effects of ginsenoside Rh2 on MCF-7 human breast cancer cells. Int J Oncol.

[19] Li B, Zhao J, Wang CZ, Searle J, He TC, Yuan CS, Du W (2011). Ginsenoside Rh2 induces apoptosis and paraptosis-like cell death in colorectal cancer cells through activation of p53. Cancer Lett.

[20] Liu J, Shiono J, Shimizu K, Yu H, Zhang C, Jin F, Kondo R (2009). 20(R)-ginsenoside Rh2, not 20(S), is a selective osteoclastgenesis inhibitor without any cytotoxicity. Bioorg Med Chem Lett.

[21] Tu Y, Wang ZX, Shi Y (1996). An efficient asymmetric epoxidation method for trans-olefins mediated by a fructose-derived ketone. Journal of the American Chemical Society.

[22] Kasai R, Hara K, Dokan R, Suzuki N, Mizutare T, Yoshihara S, Yamasaki K (2000). Major metabolites of ginseng sapogenins formed by rat liver microsomes. Chemical and pharmaceutical bulletin-Tokyo-.

[23] Errico A (2014). Prevention: daily aspirin and chemoprevention. Nat Rev Clin Oncol.

[24] Stock AM, Troost G, Niggemann B, Zanker KS, Entschladen F (2013). Targets for anti-metastatic drug development. Curr Pharm Des.

[25] Wong VK, Cheung SS, Li T, Jiang ZH, Wang JR, Dong H, Yi XQ, Zhou H, Liu L (2010). Asian ginseng extract inhibits in vitro and in vivo growth of mouse lewis lung carcinoma via modulation of ERK-p53 and NF-kappaB signaling. J Cell Biochem.

[26] Liang X, Wang JR, Wong KW, Hsiao WL, Zhou H, Jiang ZH, Kam KT, Liu L (2014). Optimization of 2-dimensional gel electrophoresis for proteomic studies of solid tumor tissue samples. Mol Med Rep.

[27] Kam KT, Liang X, Wang JR, Wong KW, Hsiao WL, Zhou H, Jiang ZH, Liu L (2013). Evaluation on the effect of different in-gel peptide isoelectric focusing parameters in global proteomic profiling. Anal Biochem.

[28] Diaz-Ramos A, Roig-Borrellas A, Garcia-Melero A, Lopez-Alemany R (2012). alpha-Enolase, a multifunctional protein: its role on pathophysiological situations. J Biomed Biotechnol.

[29] Langer HF, Chung KJ, Orlova VV, Choi EY, Kaul S, Kruhlak MJ, Alatsatianos M, DeAngelis RA, Roche PA, Magotti P, Li X, Economopoulou M, Rafail S (2010). Complement-mediated inhibition of neovascularization reveals a point of convergence between innate immunity and angiogenesis. Blood.

[30] Misra UK, Pizzo SV (2015). Activated alpha2-Macroglobulin Binding to Human Prostate Cancer Cells Triggers Insulin-like Responses. J Biol Chem.

[31] Miceli C, Tejada A, Castaneda A, Mistry SJ (2013). Cell cycle inhibition therapy that targets stathmin in in vitro and in vivo models of breast cancer. Cancer Gene Ther.

[32] Castro MA, Dal-Pizzol F, Zdanov S, Soares M, Muller CB, Lopes FM, Zanotto-Filho A, da Cruz Fernandes M, Moreira JC, Shacter E, Klamt F (2010). CFL1 expression levels as a prognostic and drug resistance marker in nonsmall cell lung cancer. Cancer.

[33] Zhang B (2006). Rho GDP dissociation inhibitors as potential targets for anticancer treatment. Drug Resist Updat.

[34] Nie D, Lamberti M, Zacharek A, Li L, Szekeres K, Tang K, Chen Y, Honn KV (2000). Thromboxane A(2) regulation of endothelial cell migration, angiogenesis, and tumor metastasis. Biochem Biophys Res Commun.

[35] Wang Y, Tong Y, Tso PH, Wong YH (2013). Regulator of G protein signaling 19 suppresses Ras-induced neoplastic transformation and tumorigenesis. Cancer Lett.

[36] Ma W, Wong CC, Tung EK, Wong CM, Ng IO (2012). RhoE is frequently down-regulated in hepatocellular carcinoma (HCC) and suppresses HCC invasion through antagonizing the Rho/Rho-kinase/myosin phosphatase target pathway. Hepatology.

[37] Derycke LD, Bracke ME (2004). N-cadherin in the spotlight of cell-cell adhesion, differentiation, embryogenesis, invasion and signalling. Int J Dev Biol.

[38] Yamasaki S, Sakata-Sogawa K, Hasegawa A, Suzuki T, Kabu K, Sato E, Kurosaki T, Yamashita S, Tokunaga M, Nishida K, Hirano T (2007). Zinc is a novel intracellular second messenger. J Cell Biol.

[39] St-Pierre Y, Campion CG, Grosset AA (2012). A distinctive role for galectin-7 in cancer ?. Front Biosci (Landmark Ed).

[40] Hsiao KC, Shih NY, Fang HL, Huang TS, Kuo CC, Chu PY, Hung YM, Chou SW, Yang YY, Chang GC, Liu KJ (2013). Surface alpha-enolase promotes extracellular matrix degradation and tumor metastasis and represents a new therapeutic target. PLoS One.

[41] Lunt SY, Muralidhar V, Hosios AM, Israelsen WJ, Gui DY, Newhouse L, Ogrodzinski M, Hecht V, Xu K, Acevedo PN, Hollern DP, Bellinger G, Dayton TL (2015). Pyruvate kinase isoform expression alters nucleotide synthesis to impact cell proliferation. Mol Cell.

[42] Budirahardja Y, Gonczy P (2009). Coupling the cell cycle to development. Development.

[43] Xu K, Belunis C, Chu W, Weber D, Podlaski F, Huang KS, Reed SI, Vassilev LT (2003). Protein-protein interactions involved in the recognition of p27 by E3 ubiquitin ligase. Biochem J.

[44] Sanli T, Steinberg GR, Singh G, Tsakiridis T (2014). AMP-activated protein kinase (AMPK) beyond metabolism: a novel genomic stress sensor participating in the DNA damage response pathway. Cancer Biol Ther.

[45] Budanov AV (2014). The role of tumor suppressor p53 in the antioxidant defense and metabolism. Subcell Biochem.

[46] Hershko DD (2008). Oncogenic properties and prognostic implications of the ubiquitin ligase Skp2 in cancer. Cancer.

[47] Yung Y, Walker JL, Roberts JM, Assoian RK (2007). A Skp2 autoinduction loop and restriction point control. J Cell Biol.

[48] Chan CH, Morrow JK, Li CF, Gao Y, Jin G, Moten A, Stagg LJ, Ladbury JE, Cai Z, Xu D, Logothetis CJ, Hung MC, Zhang S (2013). Pharmacological inactivation of Skp2 SCF ubiquitin ligase restricts cancer stem cell traits and cancer progression. Cell.

[49] Lawan A, Zhang L, Gatzke F, Min K, Jurczak MJ, Al-Mutairi M, Richter P, Camporez JP, Couvillon A, Pesta D, Roth Flach RJ, Shulman GI, Bennett AM (2015). Hepatic mitogen-activated protein kinase phosphatase 1 selectively regulates glucose metabolism and energy homeostasis. Mol Cell Biol.

[50] Martin SK, Banuelos CA, Sadar MD, Kyprianou N (2014). N-terminal targeting of androgen receptor variant enhances response of castration resistant prostate cancer to taxane chemotherapy. Mol Oncol.

[51] Burma S, Chen BP, Murphy M, Kurimasa A, Chen DJ (2001). ATM phosphorylates histone H2AX in response to DNA double-strand breaks. J Biol Chem.

[52] Shiloh Y (2003). ATM and related protein kinases: safeguarding genome integrity. Nat Rev Cancer.

[53] Wang W, Wang H, Rayburn ER, Zhao Y, Hill DL, Zhang R (2008). 20(S)-25-methoxyl-dammarane-3beta, 12beta, 20-triol, a novel natural product for prostate cancer therapy: activity in vitro and in vivo and mechanisms of action. Br J Cancer.

[54] Chang YS, Seo EK, Gyllenhaal C, Block KI (2003). Panax ginseng: a role in cancer therapy?. Integr Cancer Ther.

[55] Jeong KJ, Kim GW, Chung SH (2014). AMP-activated protein kinase: An emerging target for ginseng. J Ginseng Res.

[56] Capello M, Ferri-Borgogno S, Cappello P, Novelli F (2011). alpha-Enolase: a promising therapeutic and diagnostic tumor target. FEBS J.

[57] Wygrecka M, Marsh LM, Morty RE, Henneke I, Guenther A, Lohmeyer J, Markart P, Preissner KT (2009). Enolase-1 promotes plasminogen-mediated recruitment of monocytes to the acutely inflamed lung. Blood.

[58] Vander Heiden MG, Cantley LC, Thompson CB (2009). Understanding the Warburg effect: the metabolic requirements of cell proliferation. Science.

[59] Baumann F, Leukel P, Doerfelt A, Beier CP, Dettmer K, Oefner PJ, Kastenberger M, Kreutz M, Nickl-Jockschat T, Bogdahn U, Bosserhoff AK, Hau P (2009). Lactate promotes glioma migration by TGF-beta2-dependent regulation of matrix metalloproteinase-2. Neuro Oncol.

[60] Zaugg K, Yao Y, Reilly PT, Kannan K, Kiarash R, Mason J, Huang P, Sawyer SK, Fuerth B, Faubert B, Kalliomaki T, Elia A, Luo X (2011). Carnitine palmitoyltransferase 1C promotes cell survival and tumor growth under conditions of metabolic stress. Genes Dev.

[61] Samudio I, Harmancey R, Fiegl M, Kantarjian H, Konopleva M, Korchin B, Kaluarachchi K, Bornmann W, Duvvuri S, Taegtmeyer H, Andreeff M (2010). Pharmacologic inhibition of fatty acid oxidation sensitizes human leukemia cells to apoptosis induction. J Clin Invest.

[62] Jin L, Li D, Alesi GN, Fan J, Kang HB, Lu Z, Boggon TJ, Jin P, Yi H, Wright ER, Duong D, Seyfried NT, Egnatchik R (2015). Glutamate Dehydrogenase 1 Signals through Antioxidant Glutathione Peroxidase 1 to Regulate Redox Homeostasis and Tumor Growth. Cancer Cell.

[63] Feng Z, Levine AJ (2010). The regulation of energy metabolism and the IGF-1/mTOR pathways by the p53 protein. Trends Cell Biol.

[64] Chan CH, Li CF, Yang WL, Gao Y, Lee SW, Feng Z, Huang HY, Tsai KK, Flores LG, Shao Y, Hazle JD, Yu D, Wei W (2012). The Skp2-SCF E3 ligase regulates Akt ubiquitination, glycolysis, herceptin sensitivity, and tumorigenesis. Cell.

[65] Lane AN, Fan TW (2015). Regulation of mammalian nucleotide metabolism and biosynthesis. Nucleic Acids Res.

[66] Tong X, Zhao F, Thompson CB (2009). The molecular determinants of de novo nucleotide biosynthesis in cancer cells. Curr Opin Genet Dev.

[67] Shiloh Y, Ziv Y (2013). The ATM protein kinase: regulating the cellular response to genotoxic stress, and more. Nat Rev Mol Cell Biol.

[68] Snima KS, Pillai P, Cherian AM, Nair SV, Lakshmanan VK (2014). Anti-diabetic drug metformin: challenges and perspectives for cancer therapy. Curr Cancer Drug Targets.

[69] Ben Sahra I, Le Marchand-Brustel Y, Tanti JF, Bost F (2010). Metformin in cancer therapy: a new perspective for an old antidiabetic drug?. Mol Cancer Ther.

[70] Eikawa S, Nishida M, Mizukami S, Yamazaki C, Nakayama E, Udono H (2015). Immune-mediated antitumor effect by type 2 diabetes drug, metformin. Proc Natl Acad Sci U S A.

[71] Vujic I, Sanlorenzo M, Posch C, Esteve-Puig R, Yen AJ, Kwong A, Tsumura A, Murphy R, Rappersberger K, Ortiz-Urda S (2014). Metformin and trametinib have synergistic effects on cell viability and tumor growth in NRAS mutant cancer. Oncotarget.

[72] Georges E, Bonneau AM, Prinos P (2011). RNAi-mediated knockdown of alpha-enolase increases the sensitivity of tumor cells to antitubulin chemotherapeutics. Int J Biochem Mol Biol.

[73] Wong VK, Chiu P, Chung SS, Chow LM, Zhao YZ, Yang BB, Ko BC (2005). Pseudolaric acid B, a novel microtubule-destabilizing agent that circumvents multidrug resistance phenotype and exhibits antitumor activity in vivo. Clin Cancer Res.

[74] Zhang W, Tan S, Paintsil E, Dutschman GE, Gullen EA, Chu E, Cheng YC (2011). Analysis of deoxyribonucleotide pools in human cancer cell lines using a liquid chromatography coupled with tandem mass spectrometry technique. Biochem Pharmacol.

