# Supplementary material for: Rh2E2, a novel metabolic suppressor, specifically inhibits energy-based metabolism of tumor cells
Source: Oncotarget. 2016 Jan 18;7(9):9907–24. doi: 10.18632/oncotarget.6934 (PMC4891092; doi:10.18632/oncotarget.6934)
Supplement: Supplementary file 1 [file oncotarget-07-09907-s001.pdf]

SUPPLEMENTARY EXPERIMENTAL PROCEDURES

IDENTIFICATION OF RH2E2

3-O-*β*-D-glucopyranosyl 20*R*,24*S*-epoxydammarane-3*β*,12*β*-triol, and its 24*R* epimer: White amorphous powder, optical rotation $[\alpha]_D^{20}$ +5.29 (*C* = 0.32, MeOH). High Resolution-ESI-MS (Positive ion mode): *m/z* 639.4480 $[M+H]^+$ (calculated for $C_{36}H_{63}O_9$: 639.4467). ^{1}H-NMR (400 MHz, C_5D_5N) δ: 5.04 (2H, d, *J* = 7.8 Hz, H-1′), 4.61 (2H, d, *J* = 11.4 Hz, H-6′a), 4.42 (2H, dd, *J* = 11.4, 5.5 Hz, H-6′b), 4.39 (2H, m, H-3′), 4.23 (2H, m, H-4′), 4.20 (2H, m H-2′), 4.15 (2H, m, H-5′), 4.12 (2H, m, H-24), 3.88 (2H, m, H-12), 3.48 (2H, dd, *J* = 11.7, 4.3, H-3), 1.61, 1.60 (3H each, s, H-27), 1.58, 1.56 (3H each, s, H-21), 1.41, 1.40 (3H each, s, H-26), 1.40, 1.39 (3H each, s, H-18), 1.10 (6H, s, H-28), 1.08, 1.07 (3H each, s, H-30), 1.06, 1.00 (3H each, s, H-19), 0.90 (6H, s, H-29). ^{13}C-NMR (100 MHz, C_5D_5N) δ: 39.7 (C-1), 27.2 (C-2), 89.3 (C-3), 40.2 (C-4), 56.9 (C-5), 19.0 (C-6), 35.8 (C-7), 40.6 (C-8), 50.8 and 50.7 (C-9), 37.6 (C-10), 32.0 and 31.9 (C-11), 71.3 (C-12), 50.2 and 50.1 (C-13), 52.2 and 52.1 (C-14), 32.1 (C-15, 24*S*-epimer), 31.7 (C-15, 24*R*-epimer), 27.3 (C-16, 24*S*-epimer), 27.0 (C-16, 24*R*-epimer), 51.4 and 51.3 (C-17), 17.0 and 16.9 (C-18), 16.3 and 16.2 (C-19), 86.8 and 86.7 (C-20), 21.9 (C-21, 24*R*-epimer), 19.6 (C-21, 24*S*-epimer), 39.8 (C-22, 24*R*-epimer), 38.7 (C-22, 24*S*-epimer), 27.5 and 27.4 (C-23), 87.6 (C-24, 24*S*-epimer) 86.4 (C-24, 24*R*-epimer), 71.1 (C-25, 24*S*-epimer), 70.7 (C-25, 24*R*-epimer), 26.6 and 26.5 (C-26), 28.0 (C-27, 24*R*-epimer), 27.6 (C-27, 24*S*-epimer), 28.7 (C-28), 17.4 (C-29), 17.7 and 17.6 (C-30), 107.4 (C-1′), 76.3 (C-2′), 79.3 (C-3′), 72.4 (C-4′), 78.9 (C-5′), 63.6 (C-6′). In addition, ^{1}H-NMR spectrum showed an anomeric proton at δ 5.04 (d, *J* = 7.8 Hz), three oxygenated methane proton signals at δ 4.12 (m), 3.88 (m) and 3.48 (dd, *J* = 11.7, 4.3), and sixteen quaternary methyl groups at δ 1.61, 1.60, 1.58, 1.56, 1.41, 1.40 (6H), 1.39, 1.10 (6H), 1.08, 1.07, 1.06, 1.00, 0.90 (6H). Furthermore, ^{13}C-NMR spectrum revealed that some signals are in pair indicating the existence of a pair of epimers. The carbon signals at δ 107.4 (C-1′), 76.3 (C-2′), 79.3 (C-3′), 72.4 (C-4′), 78.9 (C-5′), 63.6 (C-6′) were assigned to the *β*-glucopyranosyl moiety of Rh2E2. The other carbon signals were almost identical to those of 20*R*, 24*S*-epoxy-dammarane-3*β*-12*β*, 25-triol and its 24*R*-epimer by comparison with the literature data. The downfield shift of C-3 at δ 89.3 suggested that the glucosyl moiety is attached to C-3 of the aglycone.

Immunoprecipitation assay

PBS-washed cells were lysed with ice-cold IP buffer containing protease inhibitor using syringe, then incubated on ice for 30 min. After centrifugation at 20,000 *g* for 20 min at 4°C and the supernatant was collected. 200 µg proteins were pre-cleared by incubating with bead slurry with gentle agitation. After that, equal amounts of lysate proteins were incubated with protein A/G-agarose and desired primary antibody at 4°C for overnight with gentle rotation. Pulled down immune complexes with protein A/G-agarose were boiled and subjected to electrophoresis followed by immune-blotting.

AOM/DSS colorectal cancer model

Male Balb/c mice (4-week-old) were purchased from Charles River Laboratory (Horsham, PA, USA), and maintained in the Animal Care Facility of Rutgers University. Housing and care of the animals were in accordance with the guidelines established by the University's Animal Research Committee consistent with the NIH Guidelines for the Care and Use of Laboratory Animals. Mice were fed with AIN-93M diet (Research diet, NJ, USA) and kept in an air-conditioned room with controlled temperature, humidity and 12 h day/night cycle. Body weight, food, and drink consumptions were monitored every other day during the experiment. An azoxymethane (AOM)/ dextran sodium sulfate (DSS)-induced colitis-associated colon carcinogenesis model was adopted to evaluate the chemopreventive of Rh2E2. Briefly, at week 2, 5-week-old Balb/c mice were subcutaneously injected with AOM (10 mg/kg, Sigma). After 1 week, 2% DSS (molecular weight: 36000 - 50000, MP Biomedicals, Santa Ana, CA, USA) was administered in the drinking water for 7 days followed by 14 days of tap water for recovery, and this cycle was repeated twice. Rh2E2 (20, 40 or 80 mg/kg) or the vehicle (PEG400: Ethanol: water = 6:1:3, v/v) was administered by gavage feeding at 0.1 mL per 20 g every other day from the first DSS cycle and throughout the experiment. The positive control drug, aspirin (50 mg/kg) was administrated the same way starting a week before AOM injection throughout the experiment. At week 13, the mice were sacrificed by CO_2 asphyxiation and the colons (from the ileocecal junction to the anal verge) were removed for imaging capture and tumor assessment. After the measurement of weight and length, the colons were cut open longitudinally along the main axis, washed with phosphate-buffered saline and then inspected. Number, size, and location of tumors in the colons were documented based on the gross examination.

LLC-1 Xenograft mouse models

Male C57BL/6J mice at the age of 6–8 weeks were obtained from the Chinese University of Hong

Kong, Hong Kong, China. Animal care and treatment procedures are conformed to the Institutional Guidelines and Animal Ordinance (Department of Health, HKSAR). Mice were randomly divided into vehicle control, Rh2E2 treatment and other control treatment groups, $n = 11–13$. The mice from all groups were subcutaneously injected with 2×10^6 of mouse Lewis lung carcinoma cells (LLC-1) to the right dorsal region. Rh2E2 was dissolved in PEG400:ethanol:saline (60% : 10% : 30%, v/v/v), and given by intraperitoneal injection at doses of 1, 5 and 10 mg/kg or administrated by oral feeding at doses of 10, 20, 40 and 80 mg/kg for continuous 21 days. 20(*S*)-Rh2, 20(*R*)-Rh2, 20(*S*)-Rg3 and 5-Fu were adopted as control drugs. In order to make sure all groups were appropriate blinded, all the experimental procedures such as subcutaneous tumor cells injection, treatment and tumor size measurement were performed by three individual persons who were blinded for animal group identity. Body weight and tumor volume (length $\times$ width^2 $\times$ 0.52) were measured every day. After 21-day treatment course, all mice were scarified and the dissected tumors were weighted and subjected to immunohistochemical analysis.

Immunohistochemistry

The dissected lung or tumor tissues were fixed and then processed into paraffin blocks for sectioning at 7-μm. Mounted tissue sections were deparaffinized in xylene, and subsequently rehydrated in graded ethanol and ddH$_2$O. Tissue sections were then treated with 20 μg/ml of Proteinase K (Roche Diagnostics, Mannheim, Germany), followed by 3% of hydrogen peroxide to block the endogenous peroxidase activity. After serum blocking, sections were incubated with antibodies such as anti-PCNA, anti-α-enolase or anti-stathmin for 1 h or suggested to POD activity assay kit according to manufacturing instruction (Roche Diagnostics), followed by 10 min incubation with *SuperPicture*™ HRP Polymer conjugate (ZYMED Lab., Invitrogen, Carlsbad, CA). After washing, slides were incubated in DAB substrate solution until the desired stain intensity was developed. The slides were then counterstained with hematoxylin, dehydrated and mounted. For necrosis detection, tumor tissue sections were stained by hematoxylin and eosin. Quantification of protein expression signal (brown colour) was counted by the total number of cells with brown immunostaining. Immunostaining images were captured by Leica DM2500 microscope.

Proteomic analysis of LLC-1 tumor tissues and cells

50 mg of frozen mouse tumor tissues were extracted using TissueLyser LT (QIAGEN, Hilden, Germany) with metal bead for 2 min at 50 Hz with urea/thiourea lysis buffer (1:10 w/v). For cell lines, the cell pellets were successively washed with ice-cold PBS and TBSS for three times and resuspended in 500 μL of urea/thiourea lysis buffer [7 M urea, 2 M thiourea, 4% (w/v) CHAPS, 30 mM Tris/HCl and protease inhibitor, pH 9.0, (GE healthcare)]. The lysates were incubated on ice for 5 min and centrifuged at 17000 *g* at 4°C for 1 hr. The supernatants were processed with 2-D Clean Up kit and re-suspended in the urea/thiourea lysis buffer for 2D-DIGE or in Dissolution buffer containing 5% SDS provided in iTRAQ Reagent 4-Plex kit (AB SCIEX) for iTRAQ experiment. The protein concentration was determined by 2-D Quant Kit and adjusted to 5 μg/μL.

For 2-DE method, 100 μg proteins were made up to 250 μL with the rehydration buffer (8 M urea, 2% (w/v) CHAPS, 0.5% (v/v) of pH 3–10 NL IPG buffer (GE Healthcare)) contained 60 mM DTT and applied to IPG strips (pH 3–10 NL, 13 cm) (GE Healthcare). The voltage hours of IEF strips were focused for approximately 60000 Vhr. The focusing IPG strips were immediately equilibrated in SDS equilibration buffer [6 M urea, 75 mM Tris-HCl (pH 8.8), 30% glycerol (v/v), 2% SDS (w/v), 0.002% (w/v) bromophenol blue] containing 10 mg/mL DTT for 15 min, and thereafter in the SDS equilibration buffer containing 25 mg/mL IAA for 15 min. After 2-DE separation, the gels were fixed for over 30 min in 40% ethanol and 10% acetic acid and stained using silver stain kit (GE Healthcare). The stained gels were then scanned with an Image Scanner III (GE Healthcare, Chalfont St. Giles, UK) and images of the spots were automatically analyzed using 2-D Elite ImageMaster Software (GE Healthcare). Experiments were performed in triplicate. Nine image gels for each group were matched. Each matched spot was numbered, and the spot volumes were normalized by total spot volume. Differences between matched spots in two groups were analyzed by Student's *t*-test, and considered significant at p-values < 0.05. Results were expressed as change (n-fold) calculated using the ratio between the highest and lowest average spot volume between Rh2E2-treated and non-treated cells.

For 2D-DIGE method, the pH of tumor tissue samples was adjusted to pH 8.5. Samples were aliquoted at 50 μg, and the pooled internal standard was made with 25 μg of each of the 12 test samples combined. Each protein samples were fluorescence labelled by incubation with 400 pmol (in 1 μL of anhydrous DMF) of CyDye (Cy3, Cy5, or Cy2) on ice for 30 min. The samples were then combined for each gel and successively incubated with 10 mM DTT and 40 mM IAA for 1 hr on ice for proper reduction and alkylation. The mixtures were made up to 450 μL with rehydration buffer and applied to Immobiline DryStrip gels (IPG strips; pH 3–10 NL, 24 cm) (GE Healthcare) by in-gel rehydration for 16 hr at

room temperature in an immobiline DryStrip Reswelling Tray (GE Healthcare). The IPG strips were transferred to an Ettan IPGphor II Manfold (GE Healthcare). IEF strips were focused for 50000 Vhr. After electrophoresis, gels were scanned directly between the glass plates using a Typhoon 9400 (GE Healthcare) laser scanner according to the manufacturer's recommendations as described before [Analytical Biochemistry Volume 443, Issue 1, 1 December 2013, Pages 27–33], variable mode imager at each of the appropriate CyDye excitation wavelengths (Cy3 (532 nm), Cy5 (633 nm), Cy2 (488 nm)). Image analysis was carried out with DeCyder differential analysis software 7.0 (GE Healthcare). Multiplexed analysis was selected for DIGE experiments and a representative reference gel image was selected for MALDI-TOF/TOF analysis

For iTRAQ labelling, the sample labelling was carried out using iTRAQ Reagent 4-Plex kit (AB SCIEX) based on the manufacturer's protocol. Briefly, the lysates were reduced and alkylated, then suggested to trypsin digestion with protein ratio of 1:10 (w/w) at 37°C for 16 hr. Six vials of iTRAQ reagent 116 was used to label mice tumor control sample while 6 vials of iTRAQ reagent 117 labeled mice tumor tissue sample with Rh2E2 treatment (iTRAQ pairs were always chosen between 114/115 or 116/117 to avoid isotopic correction during data analysis). The labelled digested peptides were incubated at room temperature for 2 hr. Subsequently, both samples were combined and taken up to a total of 1 mL with 0.1% TFA prior to cleanup on Sep-Pak C18. After isoelectric focusing, peptides from IPG strip fractions were extracted with a gradient solution (sequential extractions with 0.1% TFA, 50% ACN/0.1%TFA and 100% ACN/0.1% TFA), with gentle shaking for 1 hr each at room temperature. The eluate was then vacuum dried and cleaned up by using Waters 1 cc/10 mg OASIS HLB cartridge and eluted with 70% ACN/0.1% TFA twice. Eluates were dried under vacuum and reconstituted in 25 µL of 2% ACN/0.1% TFA before 1D-LC-MS/MS analysis. Approximately 0.2 µg of peptides were subjected to automated LC-MS/MS analysis, using Dionex Ultimate 3000 RSLC system coupled on-line to Bruker maXis Impact Q-TOF MS.

For database search and bioinformatics analysis, MS/MS data was processed using Bruker Compass Data Analysis software, and the generated peaklists were submitted to MASCOT search engine against SwissProt 51.6 database. Search parameters were defined as digestion with trypsin, fixed modification of methylthiolation at cysteine, variable modification of N-terminal acetylation of protein, conversion of N-terminal glutamine to pyro-glutamate, oxidation of methionine, and iTRAQ labeling at lysine residue and N-terminus of peptides. One missed cleavage, mass accuracy of 6 ppm on the parent ion, and 0.6 Da on fragment ions were allowed. The peptides with weight value higher than 0.5 and intensity of reporter ion higher than 20 were considered for protein quantification. The median value of ion ratios was calculated as protein ratio.

LC-MS/MS measurement of ATP metabolites

LLC-1 cells were harvested in 12 mL of ice-cold phosphate buffered saline (PBS). The cell pellet was then treated with 150 µL of 15% trichloroacetic acid (TCA) containing 7.5 µL of 20.0 µM ATP13C, 15N as internal standard and placed on ice for 10 minutes. After centrifugation at 13,500 rpm for 15 min, the acidic supernatant was separated and neutralized twice with 80 µL mixture of trioctylamine and 1, 1, 2-trichlorotrifluoroethane (a volume ratio of 45 to 55). Samples were ready for LC-MS/MS analysis. A Thermo Fisher TSQ LC–MS/MS system consisted of an Accela Autosampler, an Accela pump and a Quantum Access triple quadrupole mass spectrometer. Data acquisition was performed with the Xcalibur software version 2.0.7, and data processing was carried out using the Thermo LCquan 2.5.6 data analysis program. The chromatographic separation was achieved on a XTerra-MS C18 column (150 mm × 2.1 mm i.d., 3.5 µm, Waters, Milford, MA). The two eluents were: (A) 5 mM HA–0.5% DEA in water, pH adjusted to 10 with acetic acid; and (B) 50% acetonitrile in water. The mobile phase consisted of linear gradients of A and B: 0–15 min, 100–80% A (v/v); 15–35 min, 80–70% A; 35–45 min, 70–45% A; 45–46 min, 45–0% A; 46–50 min, 0–0% A; 51–70 min, 100–100% A. The liquid flow-rate was set at 0.3 mL/min, and the column temperature was maintained at 35 ºC.

Cell invasion assay

The cancer cell invasion assay was performed in a Cell Invasion Chamber, a 24-well tissue culture plate with cell culture inserts that contain an 8 µm pore size polycarbonate membrane over a thin layer of dried ECMatrix™ (CHEMICON). H1299 cells (15000 cells/well) were re-suspended in serum-free medium and incubated in an invasion chamber insert with different concentrations of Rh2E2 for 72 h, while the lower chamber contained medium with 10% FBS. The cells invaded through the ECM layer to the bottom of the polycarbonate membrane were labeled with Cell Stain provided in the kit for 20 min at room temperature. The non-invading cells were gently removed from the interior of the inserts by using a cotton-tipped swab. The number of invaded cells was counted through the microscope and quantified by dissolving stained cells in 10% acetic acid (200 µL/well). The colorimetric reading of the solute mixture was determined by spectrophotometer at OD 560 nm.

Acetyl-CoA assay

LLC-1 cells were treated with 80 µM Rh2E2 for 24 h. The cell lysates were then harvested for determination of acetyl-coenzyme A (Acetyl-CoA) by Acetyl-CoA Assay Kit (Sigma, MO, USA) following manufacturer's instruction. In brief, 2×10^6 Rh2E2-treated LLC-1 cancer cells were homogenized and deproteinized with 2 µL of 1 N perchloric acid/mg of sample on ice. The cell lysates were centrifuged at 13,000 × g for 10 minutes to remove insoluble material. The supernatant was then neutralized with 3 M potassium bicarbonate solution, adding in aliquots of 1 µL/10 µL of supernatant while vortexing until bubble evolution ceases. The samples were cool on ice for 5 minutes and then mcentrifuged to pellet potassium bicarbonate. Bring samples to a final volume of 50 µL with Acetyl-CoA Assay Buffer, mixed and incubated the reaction in the well for 10 minutes at 37°C at dark. After incubation, the absorbance of fluorescence intensity (λex = 535/λem = 587 nm) was detected by the TECAN plate reader and the concentration of Acetyl-CoA was calculated based on the standard curve. The calculation formula is as follow,

Concentration = S_a/S_v

S_a = Amount of Acetyl-CoA in unknown sample (pmole) from standard curve

S_v = Sample volume added into the wells

α-Ketoglutarate assay

LLC-1 cancer cells treated with or without 80 µM of Rh2E2 were harvested for determination of α-Ketoglutarate (α-KG) by α-KG Assay Kit (Sigma, MO, USA) following manufacturer's instruction. In brief, 2 × 10^6 LLC-1 cells were homogenized and deproteinized with 10kDa MWCO spin filter. The cell lysates were then mixed with α-KG assay reagent and incubated in 96 well plate for 30 minutes at 37°C. After that, the mixture absorbance at 570 nm (A570) was detected by the TECAN plate reader and the concentration of α-KG was calculated based on the standard curve. The calculation formula is as follow,

Concentration = Sa /Sv,

Sa = amount of α-KG in unknown sample from standard curve

Sv = sample volume added into the wells

SUPPLEMENTARY FIGUERS AND TABLES

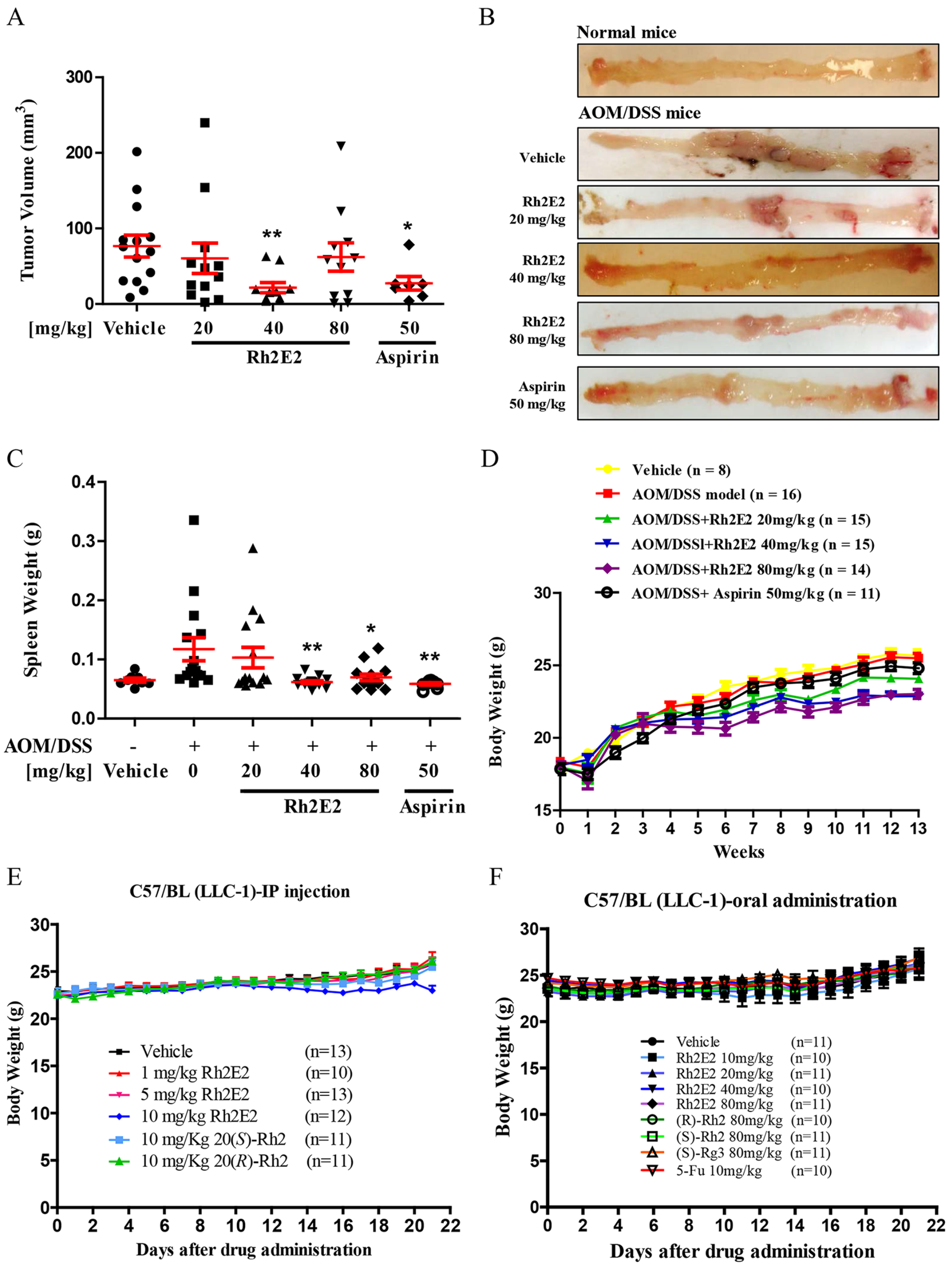

Supplementary Figure S1: *In vivo* anti-tumor effect of Rh2E2 on colitis-associated colon carcinogenesis and LLC-1 xenograft model. A. Effect of Rh2E2 on the tumor volume of neoplasms found in mice colon. After the drug treatment, the colon were dissected longitudinally and the volume of tumor in length (mm) × width (mm) × height (mm) were measured by caliper. Data were presented as mean ± SEM, $^{*}P < 0.05$, $^{**}P < 0.01$, versus AOM/DSS vehicle control group. **B.** Macroscopic view of colon in mice. Colorectal neoplasms were frequently observed in the middle and distal colon. **C.** Effect of Rh2E2 on spleen weight of AOM/DSS mice. After the mice were sacrificed, spleens were removed and weighted. Data were shown as mean ± SEM, $^{*}P < 0.05$, $^{**}P < 0.01$, versus AOM/DSS group. **D.** Effect of Rh2E2 on body weight of AOM/DSS mice. All mice body weight was recorded on the first day of each week for a total of 13 weeks. Data were expressed as mean ± SEM. **E.** Body weight change of mice with intraperitoneal injection of Rh2E2. **F.** Body weight change of mice with oral administration of Rh2E2.

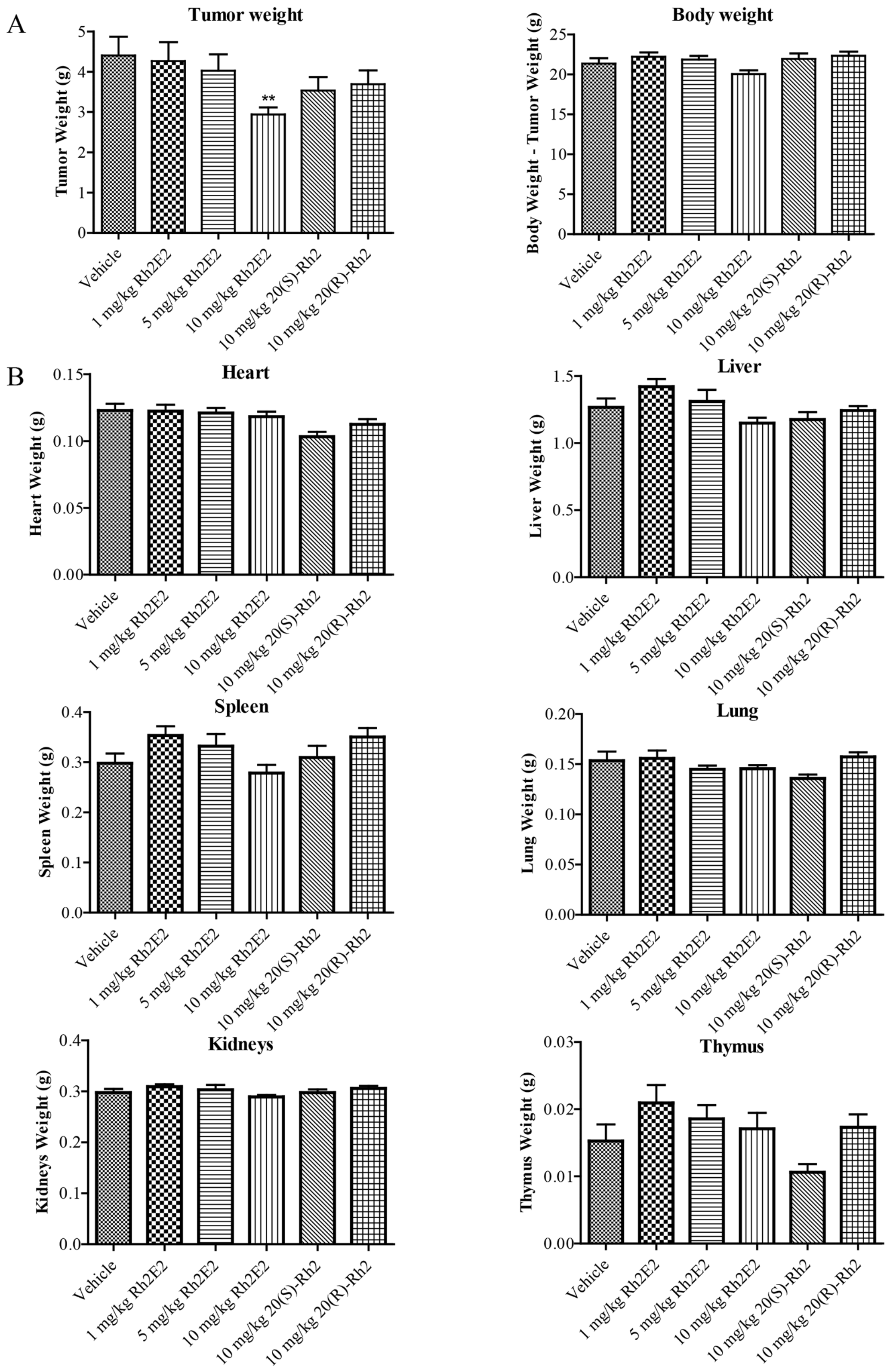

Supplementary Figure S2: The tumor suppression effect of Rh2E2 in LLC-1 xenograft model via ip injection. A. The tumor weight and body weight change of mice after Rh2E2 treatment. **B.** The organs weight change of mice after Rh2E2 treatment.

A

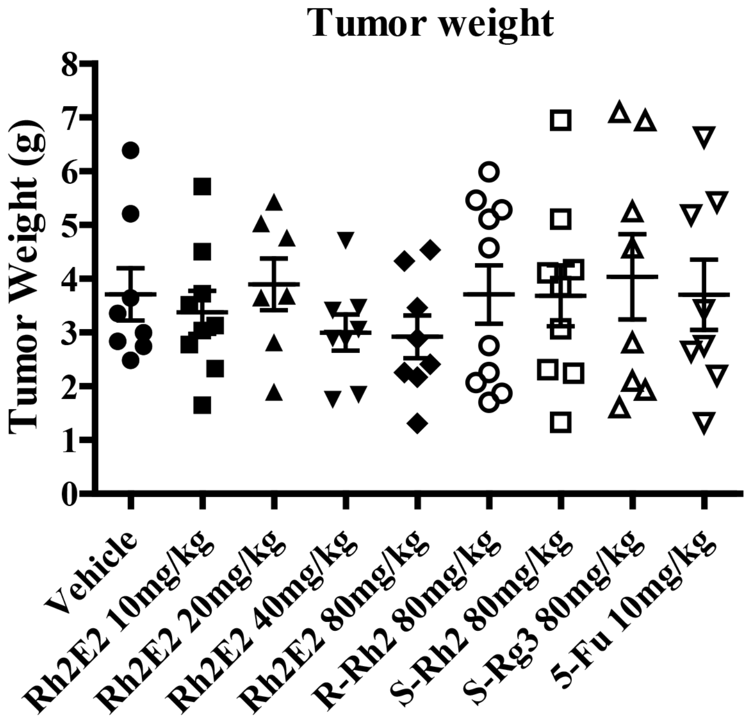

B

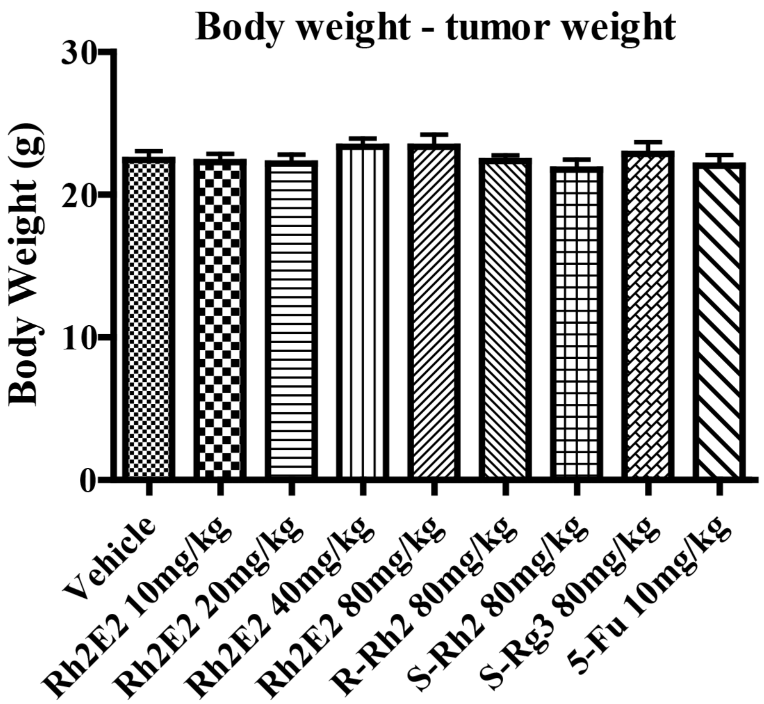

Supplementary Figure S3: The tumor suppression effect of Rh2E2 in LLC-1 xenograft model via oral administration. **A.** The tumor weight change of mice after Rh2E2 treatment. **B.** The body weight change of mice after Rh2E2 treatment.

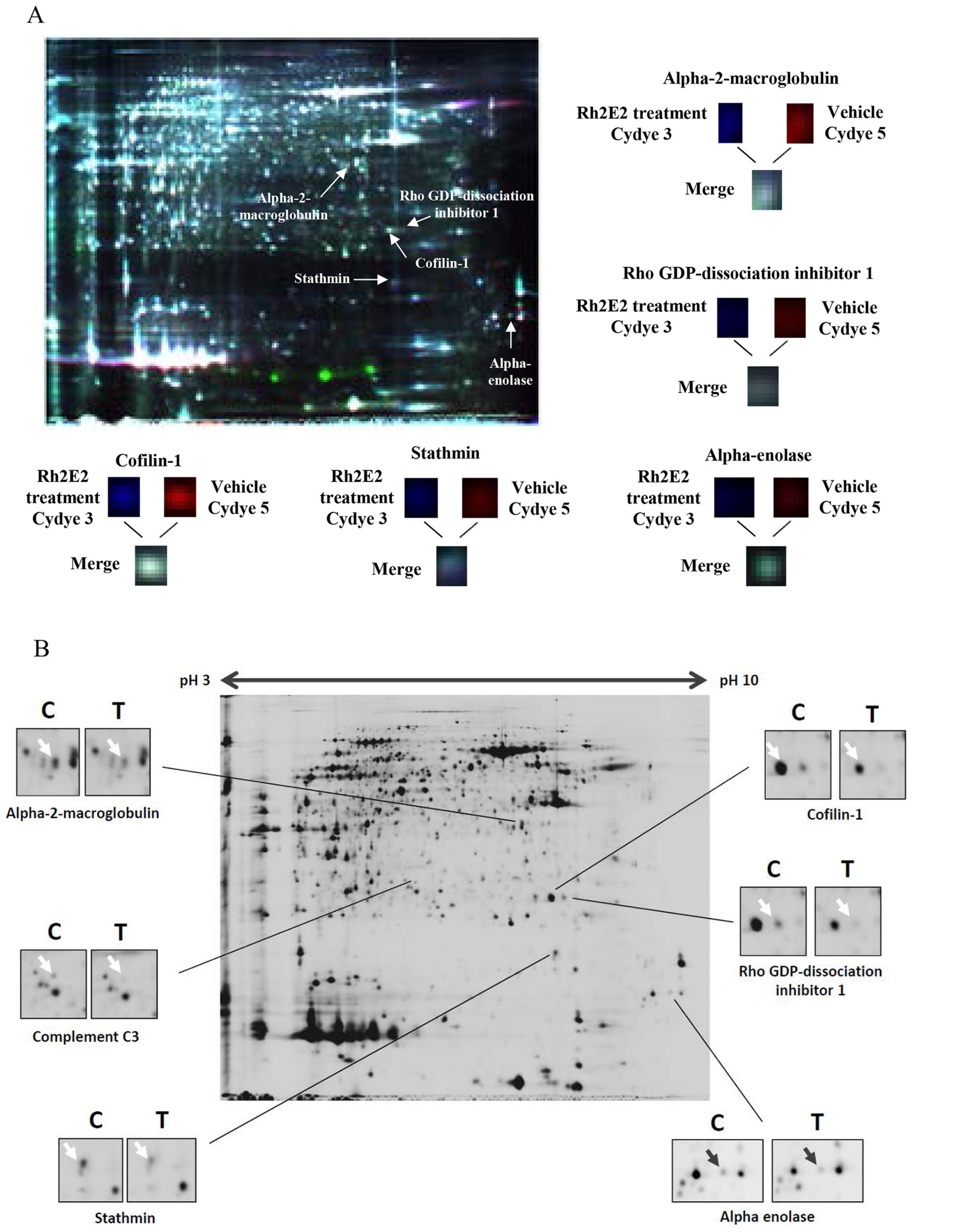

Supplementary Figure S4: Proteomic analysis of tumor tissues from vehicle control or Rh2E2-treated LLC-1 xenograft. A. Differentially expressed proteins including α-2-macroglobulin, Rho GDP-dissociation inhibitor 1, cofilin-1, stathmin and α-enolase were identified by 2D-DIGE. **B.** The differentially expressed protein spots were visualized in stained 2D-gel.

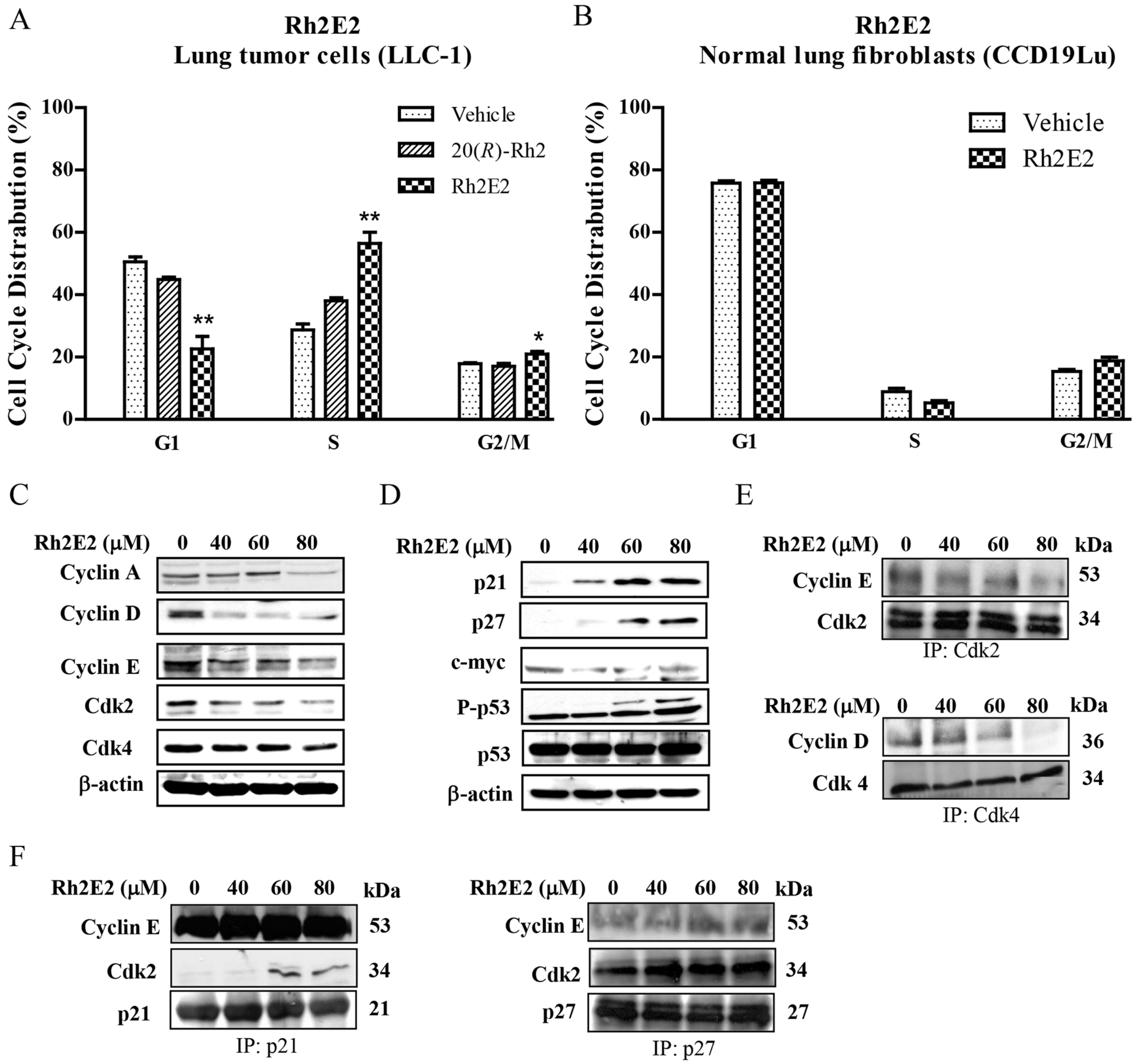

Supplementary Figure S5: Rh2E2 induced S-phase cell cycle arrest through regulation of Cdks/Cyclins and Cdks inhibitors expression. A. Effects of 20(*R*)-Rh2 and Rh2E2 on cell cycle progression in LLC-1 cancer cells. **B.** Effect of Rh2E2 on cell cycle progression in CCD19Lu normal lung cells. Exponentially growing LLC-1 or CCD19Lu cells were synchronized in the serum-free medium for 24 h. Then the cells were incubated with the DMSO, 80 μM 20(*R*)-Rh2 or 80 μM Rh2E2 for 48 hr. The cell cycle progression was evaluated using propidium iodide staining and flow cytometry analysis. The bar chart indicated the results of quantitative analysis of cell-cycle distribution (% of cell population). Means ± S.E.M. were from three independent experiments (One-way ANOVA: $^{*}P < 0.05$, $^{**}P < 0.01$). **C.** Effect of Rh2E2 on expression level of S phase specific cdks/cyclins. **D.** Effect of Rh2E2 on expression of cdk inhibitors during cell cycle progression. **E.** Rh2E2 inhibited the interaction between cyclin E/cdk2 and cyclin D/cdk4. The relative amount of cyclin E/D bound to cdk2/4 was normalized to its corresponding cdk2/4 levels. **F.** The interaction between p21 or p27 with cyclin E/cdk2 in response to Rh2E2 treatment. The relative amount of cyclin E/cdk2 bound to p21 or p27 was normalized to its corresponding p21 or p27 levels.

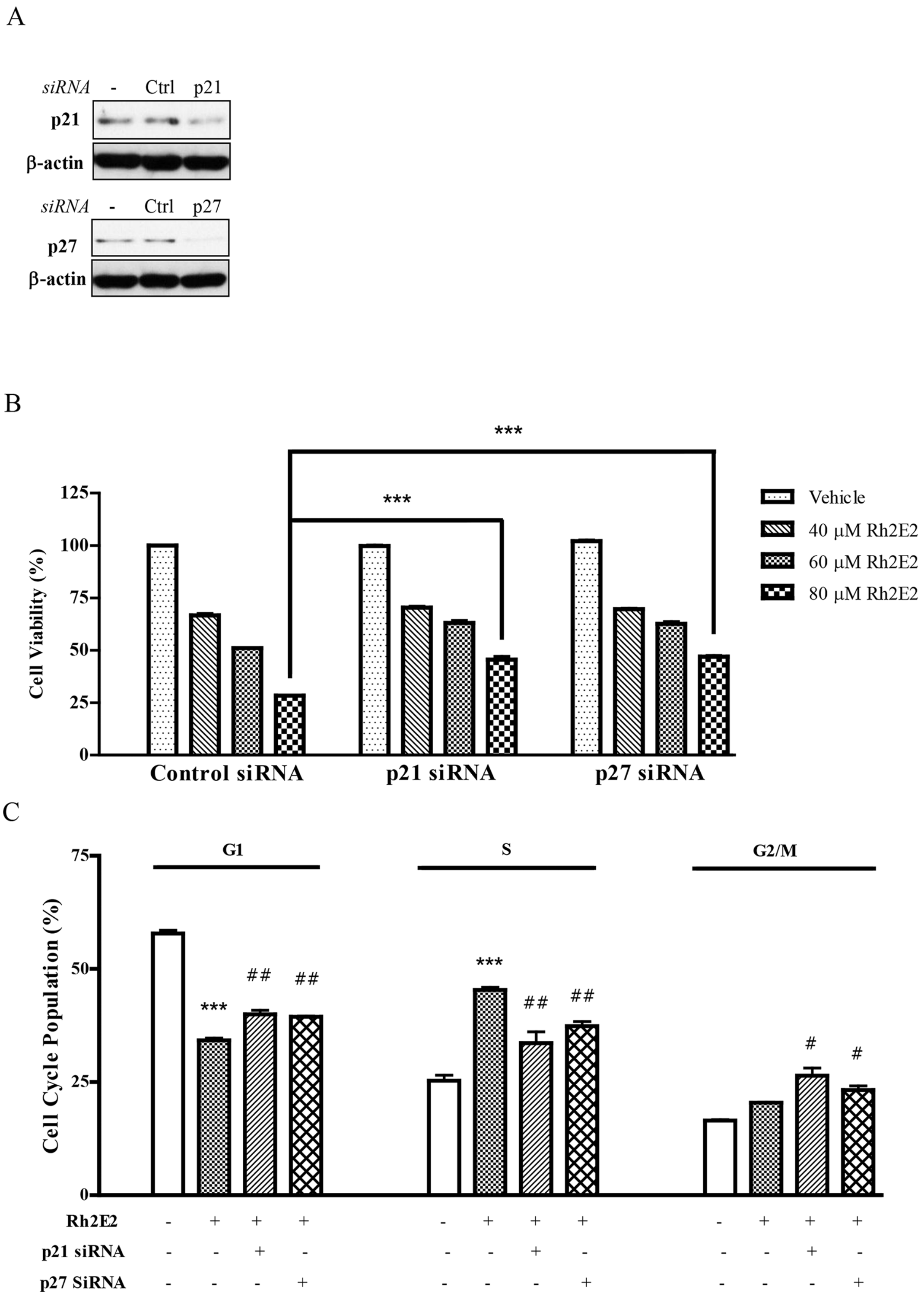

Supplementary Figure S6: Rh2E2 induced S-phase cell cycle arrest and cell death via p21 and p27. A. siRNA knockdown of p21 and p27 in LLC-1 cells. **B.** Rh2E2-mediated cell cytotoxicity was dependent on p21 and p27 expression. Cytotoxic effect of Rh2E2 in LLC-1 cells transfected with control nonspecific siRNA or p21- or p27-targeted siRNA. siRNA transfected cells were treated with DMSO (control) or Rh2E2 at indicated drug concentrations for 48 h and then subjected to MTT assay. Means ± S.E.M. were from three independent experiments (One-way ANOVA: $^{***}P < 0.001$). **C.** Cell cycle progression of LLC-1 cells transfected with the control nonspecific *si*RNA or p21- or p27-targeted *si*RNA. *si*RNA transfected cells were treated with DMSO (control) or 80 µM Rh2E2 for 48 h and then evaluated using propidium iodide and flow cytometry analysis. Means ± S.E.M. were from three independent experiments (*t*-test: $^{***}P < 0.001$ comparing with non-transfected group; $^{\#}P < 0.05$, $^{\#\#}P < 0.01$ comparing with Rh2E2-treated group).

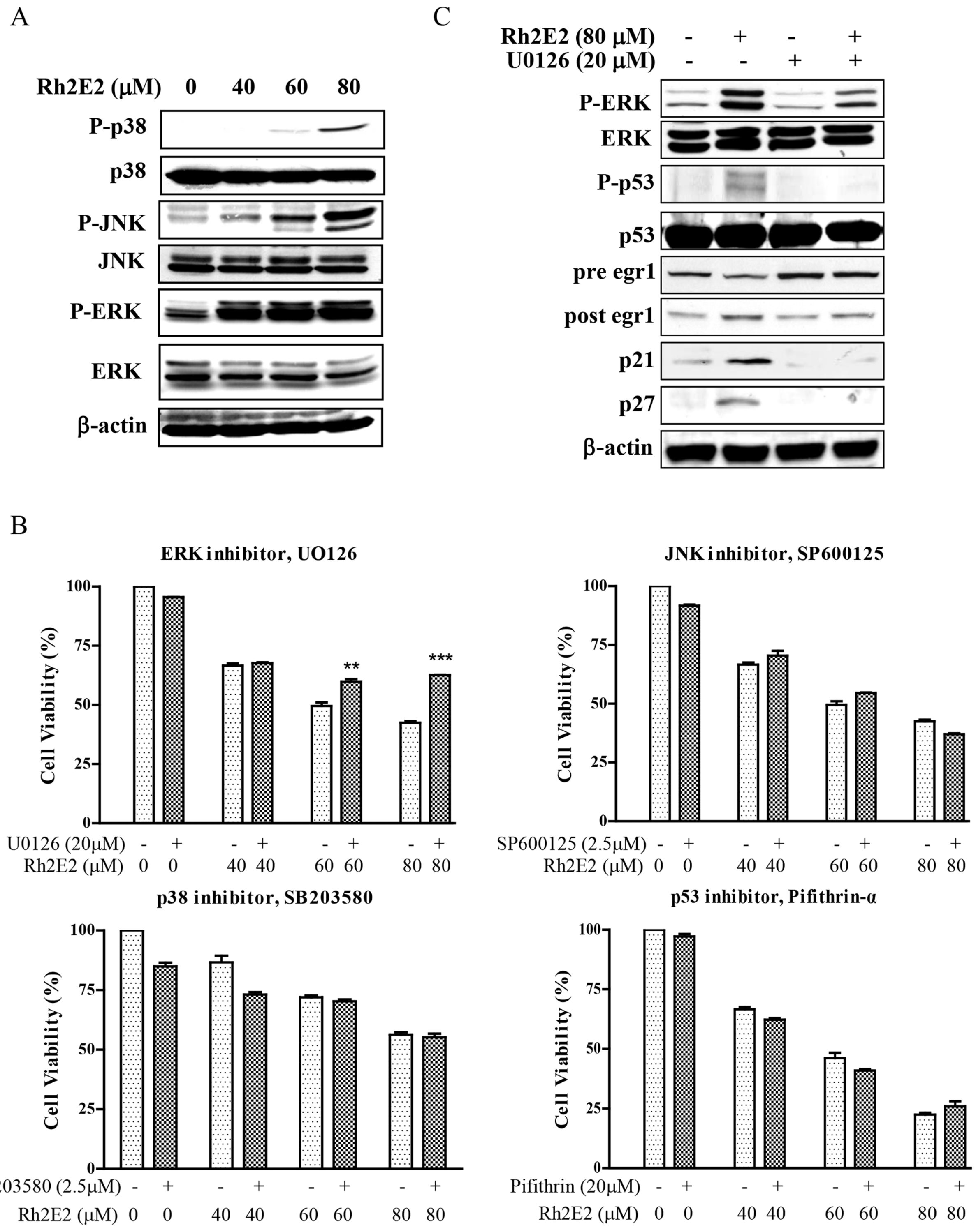

Supplementary Figure S7: Effect of Rh2E2 on MAPK signalling pathways. A. Rh2E2 activated MAPK signalling via phosphorylation of p38, p-JNK, and p-ERK. **B.** Rh2E2-mediated cell cytotoxicity through modification of MAPKs and p53 signalling. $^{**}P < 0.01$, $^{***}P < 0.001$ comparing with MAPK inhibitor free Rh2E2-treatment group. **C.** Involvement of ERK/p53 and ERK/egr1 signalling in the Rh2E2-induced p21 and p27 expression.

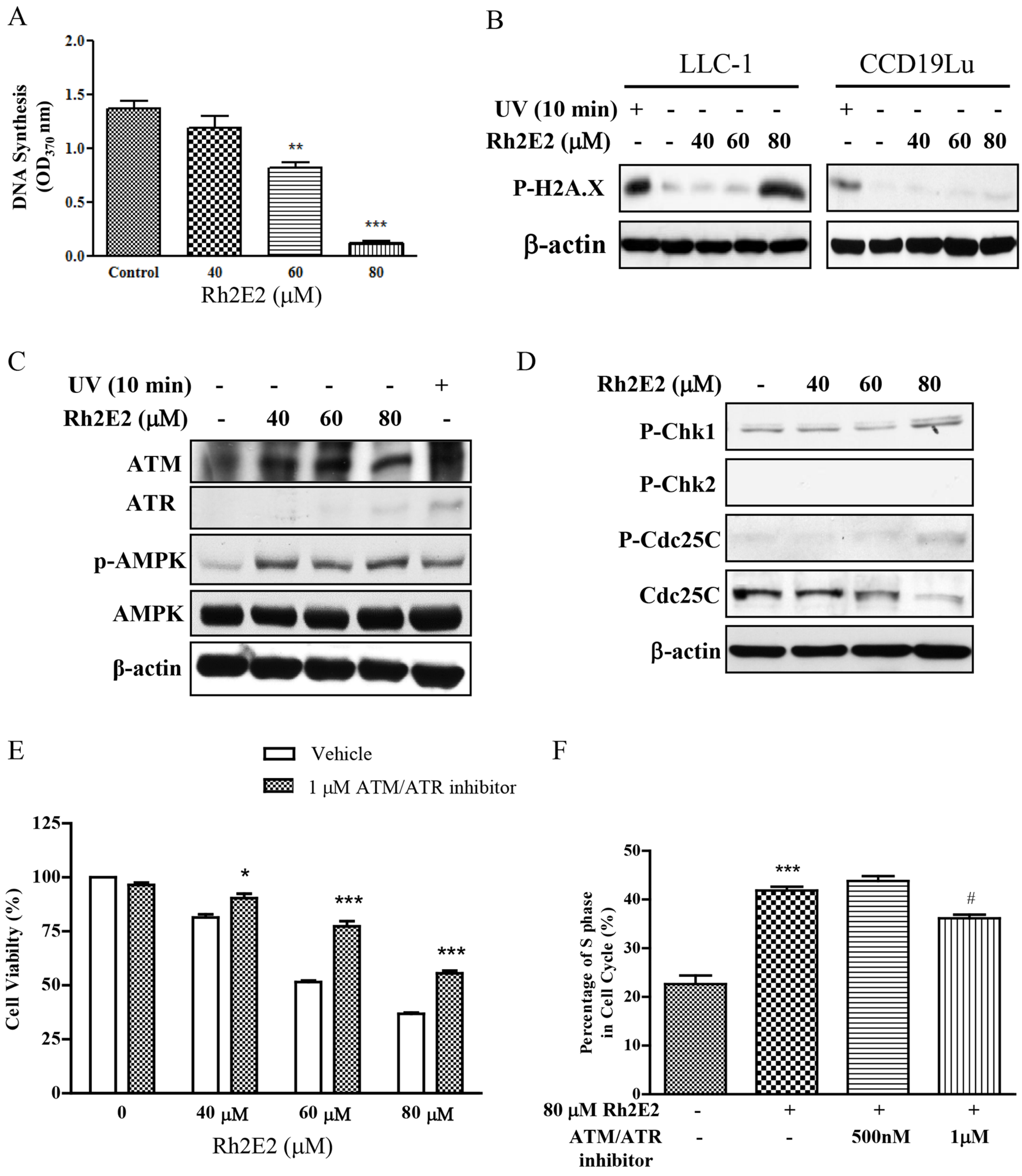

Supplementary Figure S8: Effect of Rh2E2 on genotoxic stress and ATM/ATR signalling pathways activation. A. Rh2E2 inhibited the DNA synthesis in LLC-1 cancer cells. The cells were grown in 96 well plates followed by treatment with indicated concentrations of Rh2E2 for 48 hr. The cells were then incubated with BrdU for 4 hr followed by incubation with anti-BrdU antibody and substrate by using ELISA reader. Data were presented as mean ± S.E.M. $^{**}P < 0.01$ and $^{***}P < 0.001$. **B.** Rh2E2 induced H2A.X (Ser139) phosphorylation in LLC-1 cancer cells but not normal cells. **C.** Rh2E2 activated the ATM and AMPK signalling pathways. **D.** Rh2E2 activated the Chk1/2-Cdc25C signalling pathway. LLC-1 cancer cells were treated with indicated concentrations of Rh2E2 for 48 hr. Protein was lysed and harvested with RIPA buffer. Cell lysates were separated by 10% SDS-PAGE, and then subjected to immunoblotting with antibodies against phospho-H2A.X, ATM, ATR, phospho-Chk1 (Ser345), phospho-Chk2 (Thr68), phospho-Cdc25C (Ser216) and total Cdc25C antibodies. Data were representative of three experiments. **E.** Rh2E2-mediated cell cytotoxicity was abolished by addition of ATM/ATR inhibitor. **F.** Rh2E2-mediated S-phase cell cycle arrest was partially reduced by addition of ATM/ATR inhibitor.

Supplementary Table S1: 2D-DIGE detection of differentially expressed proteins from cancerous tissues of the vehicle- and Rh2E2-treated LLC-1 bearing mice

Prot_acc	Protein name	Fold change	*T*-test
RD23B_MOUSE	RAD23 homolog B	0.43	0.018
APOA1_MOUSE	Apolipoprotein A-I precursor	0.53	0.031
CYB5_MOUSE	Cytovillin	0.53	0.049
KNG1_MOUSE	Bradykinin	0.53	0.022
ENOA_MOUSE	Alpha-enolase	0.55	0.007
PPIP1_MOUSE	Proline-serine-threonine phosphatase-interacting protein 1	0.55	0.017
A1AT4_MOUSE	Alpha-1 protease inhibitor 4	0.55	0.015
HNRPM_MOUSE	Heterogeneous nuclear ribonucleoprotein M	0.56	0.017
HSP74_MOUSE	Heat shock 70 kDa protein 4	0.562	0.018
HSP7C_MOUSE	Heat shock cognate 71 kDa protein	0.59	0.015
CO3_MOUSE	Complement C3	0.60	0.042
TCPA2_MOUSE	T-complex protein 1 subunit alpha B	0.60	0.021
ROA2_MOUSE	Heterogeneous nuclear ribonucleoproteins A2/B1	0.62	0.032
TCPZ_MOUSE	T-complex protein 1 subunit zeta	0.63	0.024
A2M_MOUSE	Alpha-2-macroglobulin	0.65	0.049
APOH_MOUSE	Apolipoprotein H	0.66	0.020
SOD2_MOUSE	Superoxide dismutase	0.66	0.022
DDAH2_MOUSE	Dimethylargininase-2	0.67	0.014
FABP5_MOUSE	Fatty acid-binding protein	0.68	0.036
HEMO_MOUSE	Hemopexin precursor	0.70	0.033
PRDX2_MOUSE	Peroxiredoxin-2	0.71	0.012
STMN1_MOUSE	Stathmin	0.71	0.043
GALNS_MOUSE	N-acetylgalactosamine-6-sulfate sulfatase	0.72	0.013
CH3L3_MOUSE	Chitinase 3-like protein 3 precursor	0.72	0.047
GELS_MOUSE	Gelsolin precursor	0.74	0.010
ANXA1_MOUSE	Annexin A1	0.74	0.025
ATPB_MOUSE	ATP synthase subunit beta, mitochondrial precursor	0.74	0.026
PRDX1_MOUSE	Peroxiredoxin-1	0.77	0.043

(*Continued*)

Prot_acc	Protein name	Fold change	*T*-test
TCPG_MOUSE	T-complex protein 1 subunit gamma	0.77	0.010
COF1_MOUSE	Cofilin-1	0.78	0.049
NDRG1_MOUSE	Protein NDRG1	0.78	0.041
PER3_MOUSE	Period circadian protein homolog 3	0.79	0.045
ADK_MOUSE	Adenosine kinase	0.79	0.037
GDIR_MOUSE	Rho GDP-dissociation inhibitor 1	0.79	0.044
ALDH2_MOUSE	Aldehyde dehydrogenase 2, mitochondrial	1.20	0.019
LEG1_MOUSE	Galectin-1	1.22	0.028
RS12_MOUSE	40S ribosomal protein S12	1.24	0.044
PDCD5_MOUSE	Programmed cell death protein 5	1.25	0.039
EF2_MOUSE	Elongation factor 2	1.33	0.010
APT_MOUSE	Adenine phosphoribosyltransferase	1.34	0.015
ANXA5_MOUSE	Annexin A5	1.35	0.033
CALM_MOUSE	Calmodulin	1.40	0.035
TKT_MOUSE	Transketolase	1.41	0.023
CRAMP_MOUSE	Cathelin-related antimicrobial peptide	1.45	0.044
TCTP_MOUSE	Translationally-controlled tumor protein	1.48	0.010
TALDO_MOUSE	Transaldolase	1.50	0.030
EF1A1_MOUSE	Elongation factor 1-alpha 1	1.51	0.040
RSSA_MOUSE	40S ribosomal protein SA	1.86	0.043

Supplementary Table S2: iTRAQ detection of differentially expressed proteins from the cancerous tissues of the vehicle- and Rh2E2-treated LLC-1 bearing mice

Prot_acc	Prot_desc	Fold change
FOLR2_MOUSE	Folate receptor beta	0.35
MYP0_MOUSE	Myelin protein P0	0.35
C163A_MOUSE	Scavenger receptor cysteine-rich type 1 protein	0.46
AP4A_MOUSE	Bis(5~-nucleosyl)-tetraphosphatase	0.46
PLP2_MOUSE	Proteolipid protein 2	0.47
MYD88_MOUSE	Myeloid differentiation primary response protein	0.48
ENAH_MOUSE	Protein enabled homolog	0.48
SF17B_MOUSE	Splicing factor, arginine/serine-rich 17B	0.48
RABP1_MOUSE	Cellular retinoic acid-binding protein 1	0.49
COCA1_MOUSE	Collagen alpha-1(XII) chain	0.52
SAMN1_MOUSE	SAM domain-containing protein SAMSN-1	0.52
CREL2_MOUSE	Cysteine-rich with EGF-like domain protein 2	0.52
AN13A_MOUSE	Ankyrin repeat domain-containing protein 13A	0.55
DHYS_MOUSE	Deoxyhypusine synthase	0.56
CF211_MOUSE	UPF0364 protein C6orf211 homolog	0.56
BORG3_MOUSE	Cdc42 effector protein 5	0.57
TCF20_MOUSE	Transcription factor 20	0.58
IBP3_MOUSE	Insulin-like growth factor-binding protein 3	0.59
ARP19_MOUSE	cAMP-regulated phosphoprotein 19	0.60
ECHD1_MOUSE	Enoyl-CoA hydratase domain-containing protein 1	0.60
THAS_MOUSE	Thromboxane-A synthase	0.60
SPY4_MOUSE	Protein sprouty homolog 4	0.60
GGCT_MOUSE	Gamma-glutamylcyclotransferase	0.60
TAGL_MOUSE	Transgelin	0.60
P4K2B_MOUSE	Phosphatidylinositol 4-kinase type 2-beta	0.61
PTMA_MOUSE	Prothymosin alpha	0.61
USF2_MOUSE	Upstream stimulatory factor 2	0.62
GSDMC_MOUSE	Gasdermin-C	0.63
SOSSC_MOUSE	SOSS complex subunit C	0.63
E2AK4_MOUSE	Eukaryotic translation initiation factor 2-alpha kinase 4	0.64
ZC3H6_MOUSE	Zinc finger CCCH domain-containing protein 6	0.65
CHSS1_MOUSE	Chondroitin sulfate synthase 1	0.64
M11D1_MOUSE	Protein RSM22 homolog, mitochondrial	0.65
UB2D2_MOUSE	Ubiquitin-conjugating enzyme E2 D2	0.66

(*Continued*)

Prot_acc	Prot_desc	Fold change
LIPB2_MOUSE	Liprin-beta-2	0.66
NUD15_MOUSE	Probable 7,8-dihydro-8-oxoguanine triphosphatase NUDT15	0.66
CAH3_MOUSE	Carbonic anhydrase 3	0.66
CCNK_MOUSE	Cyclin-K	0.66
VTDB_MOUSE	Vitamin D-binding protein	0.66
PRTN3_MOUSE	Myeloblastin	1.50
LIPT_MOUSE	Lipoyltransferase 1, mitochondrial	1.51
RGS19_MOUSE	Regulator of G-protein signaling 19	1.52
PLCG1_MOUSE	Phosphatidylinositol bisphosphate phosphodiesterase	1.52
BL1S3_MOUSE	Biogenesis of lysosome-related organelles complex 1	1.52
MAST2_MOUSE	Microtubule-associated serine/threonine-protein kinase 2	1.52
MTUS2_MOUSE	Microtubule-associated tumor suppressor candidate 2 homolog	1.53
WIBG_MOUSE	Partner of Y14 and mago	1.53
VMAC_MOUSE	Vimentin-type intermediate filament-associated coiled-coil protein	1.53
IFIX_MOUSE	Pyrin and HIN domain-containing protein 1	1.53
SHPS1_MOUSE	Tyrosine-protein phosphatase non-receptor type substrate 1	1.54
OGFD1_MOUSE	2-oxoglutarate and iron-dependent oxygenase domain-containing protein 1	1.55
RDH13_MOUSE	Retinol dehydrogenase 13	1.55
ZFAN1_MOUSE	AN1-type zinc finger protein 1	1.56
MED12_MOUSE	Mediator of RNA polymerase II transcription	1.56
RND3_MOUSE	Rho-related GTP-binding protein RhoE	1.56
SOX3_MOUSE	Transcription factor SOX-3	1.56
RM47_MOUSE	39S ribosomal protein L47, mitochondrial	1.56
PALD_MOUSE	Paladin	1.57
ORC3_MOUSE	Origin recognition complex subunit 3	1.57
UBXN6_MOUSE	UBX domain-containing protein 6	1.57
RHBT3_MOUSE	Rho-related BTB domain-containing protein 3	1.57
CTCF_MOUSE	Transcriptional repressor CTCF	1.57
MXI1_MOUSE	Max-interacting protein 1	1.58
HSDL1_MOUSE	Inactive hydroxysteroid dehydrogenase-like protein 1	1.58
TRML1_MOUSE	Trem-like transcript 1 protein	1.58

(*Continued*)

Prot_acc	Prot_desc	Fold change
TENN_MOUSE	Tenascin-N	1.59
CADH2_MOUSE	Cadherin-2	1.59
CPNE7_MOUSE	Copine-7	1.59
K1109_MOUSE	Uncharacterized protein KIAA1109	1.59
CF125_MOUSE	Uncharacterized protein C6orf125 homolog	1.60
HAUS8_MOUSE	HAUS augmin-like complex subunit 8	1.60
PTTG_MOUSE	Pituitary tumor-transforming gene 1 protein-interacting protein	1.65
MAGG1_MOUSE	Melanoma-associated antigen G1	1.66
MTF2_MOUSE	Metal-response element-binding transcription factor 2	1.67
PIHD1_MOUSE	PIH1 domain-containing protein 1	1.71
THMS1_MOUSE	Protein THEMIS	1.72
SSRG_MOUSE	Translocon-associated protein subunit gamma	1.73
TRI69_MOUSE	Tripartite motif-containing protein 69	1.73
LEG7_MOUSE	Galectin-7	1.73
CEP70_MOUSE	Centrosomal protein of 70 kDa	1.73
CLK1_MOUSE	Dual specificity protein kinase CLK1	1.73
MIPT3_MOUSE	TRAF3-interacting protein 1	1.76
SYCE1_MOUSE	Synaptonemal complex central element protein 1	1.79
MYADM_MOUSE	Myeloid-associated differentiation marker	1.81
PILRA_MOUSE	Paired immunoglobulin-like type 2 receptor	1.84
NSD2_MOUSE	Probable histone-lysine N-methyltransferase	1.86
LIRB4_MOUSE	Leukocyte immunoglobulin-like receptor subfamily B member 4	1.87
7B2_MOUSE	Neuroendocrine protein 7B2	1.90
FBXW8_MOUSE	F-box/WD repeat-containing protein 8	1.95
GHRL_MOUSE	Appetite-regulating hormone	2.00
NDUA1_MOUSE	NADH dehydrogenase alpha subcomplex subunit 1	2.07
OSBL9_MOUSE	Oxysterol-binding protein-related protein 9	2.17
DSG2_MOUSE	Desmoglein-2	2.26
ZNT4_MOUSE	Zinc transporter 4	2.41
PA24B_MOUSE	Cytosolic phospholipase A2 beta	2.67
SUMF1_MOUSE	Sulfatase-modifying factor 1	2.71
RPTOR_MOUSE	Regulatory-associated protein of mTOR	2.86
LRIQ3_MOUSE	Leucine-rich repeat and IQ domain-containing protein 3	4.99

Supplementary Table S3: Differentially expressed mitochondrial proteins in the vehicle- and Rh2E2-treated LLC-1 lung cancer cells

Prot_acc	Protein name	Fold change	Function
ACADM_MOUSE	Medium-chain specific acyl-CoA dehydrogenase	0.527	fatty acid beta-oxidation
ECHB_MOUSE	Trifunctional enzyme subunit beta	0.596	fatty acid beta-oxidation
ACADS_MOUSE	Short-chain specific acyl-CoA dehydrogenase	0.594	fatty acid beta-oxidation
HCDH_MOUSE	Hydroxyacyl-coenzyme A dehydrogenase	0.473	fatty acid beta-oxidation
ECHM_MOUSE	Enoyl-CoA hydratase	0.449	fatty acid beta-oxidation
ETFD_MOUSE	Electron transfer flavoprotein-ubiquinone oxidoreductase	0.58	links the oxidation of fatty acids
SUCB1_MOUSE	Succinyl-CoA ligase [ADP-forming] subunit beta	0.567	tricarboxylic acid cycle
DHSA_MOUSE	Succinate dehydrogenase [ubiquinone] flavoprotein subunit	0.519	tricarboxylic acid cycle
ACON_MOUSE	Aconitate hydratase	1.687	tricarboxylic acid cycle
FUMH_MOUSE	Fumarate hydratase	0.62	tricarboxylic acid cycle
DHE3_MOUSE	Glutamate dehydrogenase 1	1.716	tricarboxylic acid cycle
ODO1_MOUSE	2-oxoglutarate dehydrogenase	0.627	tricarboxylic acid cycle
PCKGM_MOUSE	Phosphoenolpyruvate carboxykinase [GTP]	1.659	
GRP75_MOUSE	Stress-70 protein	0.469	cell proliferation
RT07_MOUSE	28S ribosomal protein S7	0.211	Ribosome biogenesis
RT15_MOUSE	28S ribosomal protein S15	0.313	Ribosome biogenesis
RT22_MOUSE	28S ribosomal protein S22	0.674	Ribosome biogenesis
RT26_MOUSE	28S ribosomal protein S26	0.406	Ribosome biogenesis
RT35_MOUSE	28S ribosomal protein S35	0.51	Ribosome biogenesis
C1QBP_MOUSE	Complement component 1 Q subcomponent-binding protein	1.595	Ribosome biogenesis
QCR2_MOUSE	Cytochrome b-c1 complex subunit 2	0.613	mitochondrial respiratory
NDUV1_MOUSE	NADH dehydrogenase [ubiquinone] flavoprotein 1	0.548	mitochondrial respiratory
NDUS1_MOUSE	NADH-ubiquinone oxidoreductase 75 kDa subunit	0.614	mitochondrial respiratory
CIA30_MOUSE	Complex I intermediate-associated protein 30	0.631	mitochondrial respiratory
ATP5H_MOUSE	ATP synthase subunit d	0.546	Hydrogen ion transport
OAT_MOUSE	Ornithine aminotransferase	0.354	Amino-acid biosynthesis
SCOT1_MOUSE	Succinyl-CoA:3-ketoacid-coenzyme A transferase 1	0.513	Ketone metabolism
STML2_MOUSE	Stomatin-like protein 2	1.624	Regulates mitochondrial function
TIM13_MOUSE	Mitochondrial import inner membrane translocase subunit Tim13	2.494	import of metabolite transporters

Supplementary Table S4: Comparison of the differentially expressed proteins from the vehicle- and Rh2E2-treated LLC-1 lung cancer cells verse vehicle and Rh2E2-treated CCD19Lu normal lung fibroblasts

Protein name	Fold Change	
	LLC-1	CCD19Lu
Medium-chain specific acyl-CoA dehydrogenase	0.527	N/A
Trifunctional enzyme subunit beta	0.596	N/A
Short-chain specific acyl-CoA dehydrogenase	0.594	N/A
Hydroxyacyl-coenzyme A dehydrogenase	0.473	N/A
Enoyl-CoA hydratase	0.449	N/A
Electron transfer flavoprotein-ubiquinone oxidoreductase	0.58	0.949
Succinyl-CoA ligase [ADP-forming] subunit beta	0.567	0.967
Succinate dehydrogenase	0.519	N/A
Aconitate hydratase	1.687	N/A
Fumarate hydratase	0.62	0.962
2-oxoglutarate dehydrogenase	0.627	0.996
Stathmin	0.65	1.378
Alpha-enolase	0.60	0.942
